# *Elsholtzia*: phytochemistry and biological activities

**DOI:** 10.1186/1752-153X-6-147

**Published:** 2012-12-05

**Authors:** Zhiqin Guo, Zizhen Liu, Xiaohong Wang, Weirui Liu, Rui Jiang, Ruiyang Cheng, Gaimei She

**Affiliations:** 1 School of Chinese Pharmacy, Beijing University of Chinese Medicine, Beijing, 100102, China

## Abstract

Plants of the genus *Elsholtzia* (Lamiaceae) have a long history of medicinal use in folk. The phytochemical investigations revealed the presence of flavonoids, phenylpropanoids, terpenoids, and other compounds. Abundant volatile components are also identified. Pure compounds, volatile constituents and crude extracts from the genus exhibited a wide spectrum of *in vitro* and *in vivo* pharmacological activities. The aims of this review hopefully provide comprehensive information on the distribution, phytochemistry, volatile components, and pharmacological research of *Elsholtzia* for exploring the potential and advance researches.

## Review

### Background

*Elsholtzia* is a genus containing at least 33 species in the family Lamiaceae. They have been widely distributed and applied in East Asia, Africa, North America, and European countries for centuries. The genus *Elsholtzia* plants are mostly aromatic plants, always used as domestic folk medicine, herbal tea, food, spices, beverages, perfumeries, cosmetics, aromatherapies, and the source of honey manufacture.

As folk medicine, the plants in the genus have been used for the treatment of colds, headaches, pharyngitis, fever, diarrhea, digestion disorder, rheumatic arthritis, nephritises, and nyctalopia in China [1-3]. Another important application of the plants is to repair soil that is contaminated by heavy metals. A growing number of research works are focusing on the function of the genus for repairing soil. The most known one is from *E*. *splendens* (*E*. *haichowensis*), which is a Cu-accumulator plant widely distributed in Cu-mining wastes and Cu-contaminated soil in China [4-7].

The paper intends to provide a significant insight into the distribution, phytochemical and pharmacological investigations and hopefully to provide preliminary data for further study and development of the clinical uses of the genus.

### Distribution

The genus *Elsholtzia* (Lamiaceae) was widely found in East Asia, Africa, North America, and Europe, especially in China, Korea, Japan, and India. So far, it is reported that at least 33 species plants of the genus are distributed in China [1,2]. And most of *Elsholtzia* live at an altitude of 1000 to 3000 meters. *E*. *splendens* survives at altitude from 200 to 300 meters. *E*. *cephalantha*, *E*. *strobilifera* and *E*. *eriostachya* grow in high altitude of 3000 to 4000 meters.

*Elsholtzia* generally exists at hilly grasslands, waste areas, forests, thickets, or valleys in warm area. *E*. *saxatilis*, named ‘Yansheng Xiangru’ in Chinese, grows in rocky crevices. And *E*. *saxatilis* is different from other plants of *Elsholtzia*, being distributed in the northeast of Asia, such as northeast of China, Korea, Russia, and Japan, where are cold compared with in South Asia. So far, *E*. *communis* and *E*. *argyi* have been cultivated in Yunnan province (China) and Vietnam. Except for the two mentioned species, most other plants in the genus are wild [1-3]. The distribution of all 33 species of *Elsholtzia* is shown in Additional file 1: Table S1.

### Chemical constituents

Previous phytochemical investigations showed that flavonoids are major ingredients in *Elsholtzia*. They are characterized by the presence of the substitutional groups and modes, as well as their glycosides. Phenylpropanoids, terpenoids, phytosterols, and cyanogenic glycosides are also main chemical constituents in this genus. In this section, we summarize and classify all reported constituents from *Elsholtzia*. Compounds **1**–**144** and the corresponding plant sources are list in Additional file 2: Table S2 and the structures **1**–**132** are showed in Additional file 3: Figure S1.

### C6-C3 constituents

Up to now, there are 68 C6-C3-C6 compounds isolated and reported from *Elsholtzia*, including flavonoids and their glycosides. Its number and content are the most in all secondary metabolites derived from the genus.

Firstly, compounds **130** are flavones, in which several hydroxyls, methoxyls and glycosyl groups are linked to the mother nucleus. The oxygenic function groups are most commonly attached to the C-5 and C-7 positions in the flavones. A small amount of 5, 6-dihydroxy-7, 8-dimethoxy flavone (**2**) from *E*. *splendens* was obtained by preparative TLC, which is the second report except its first isolation from the roots of *Scutellaria ramosissima*[8]. Luteolin 7-*O*-[6"-(3"'-hydroxy-4"'-methoxy cinnamoyl)]-β-D-glucopyranoside (**28**) and luteolin 7-*O*-(6"-feruloyl)-β-D-glucopyranoside (**29**) are very semblable in structure. The difference is that the glucose moiety C-6 position is attached to a 3"'-hydroxy-4"'-methoxy cinnamoyl in **28**, while a 6"-feruloyl in **29**. **28** was isolated from the whole plants of *E*. *bodinieri* as a new compound [9]. Secondly, 14 flavonols, **3144**, were isolated from the genus. A number of free hydroxys are attached to the C-3 positions in **3234**. Compounds **3544** are 3-*O*-flavonol glycosides, linked with diverse glycosyl groups, *i*.*e*. glucopyranosyl, galactosyl or rhamnopyranosyl. Also, four prenyl-flavonoids (**4548**) occur in *E*. *rugulosa* and *E*. *stauntonii*, among which 5, 7, 3', 4'-tetrahydroxy-5'-C-prenylflavone 7-*O*-β-D-glucoside (**46**) and muxiangrine III (**47**) were reported as new compounds [10-13]. 3"-Hydroxy-4", 5"-dimethoxyfuranoflavone (**49**) and 3", 4", 5"-trimethoxyfuranoflavone (**50**) are furanoflavone. They possess the characteristic of an unsaturated oxygen-containing furanyl fused into the ring-A's C-6 and C-7 positions. They are the two unique furanoflavones discovered in the genus so far [14]. Structurally compounds **5155** are all characterized by a gem-dimethylchromene moiety and one hydroxyl attached to C-3' and C-4' of ring-B. Muxiangrine I (**51**) and muxiangrine II (**52**) were isolated from *E*. *stauntonii*[11,12]. Compounds **5355**, named sifanghaoines I–III, were new compounds from *E*. *blanda* respectively [15,16]. 5'-Dihydroxy-7-acetoxyl-6,8,3",3"-tetramethylpyran-(3',4')flavone (**53**) and 5,5'-dihydroxy-7- (α-methy1) butyroxyl-6,8,3",3"-tetramethylpyran-(3', 4')flavone (**54**) are structurally similar. The distinguishment is that **53** is linked by an acetate group at C-7 position, while **54** is linked by a (α-methyl) butyroxyl moiety. A methylenedioxy is substituted C-6 and C-7 positions in 5,5'-dihydroxy-6,7-methylenedioxy-8,3",3"-trimethylpyran-(3',4')flavone (**55**). The same substituent ion also occurs in **47**.

Furthermore, eight flavanones (**5663**) and one flavanonol (**64**) are listed in Additional file 2: Table S2. Compounds **5862** glycosylated at C-7 position, are mainly existed in *E*. *bodinieri*[17,18]. Eriodictyol 7-*O*-(6"-feruloyl)-β-D-glucopyranoside (**59**) and eriodictyol 7-*O*-[6"-(3"'-hydroxy-4"'-methoxy cinnamoyl)]-β-D-glucopyranoside (**60**) were isolated from *E*. *bodinieri* as new flavanone glycosides [17]. The feruloyl is attached to the glucosyl C-6" position in compound **59**. Compound 60 is an isomer of **59**, with -OCH_3_ and -OH groups at the C-3"' and C-4"' positions in the 3,4-substituted coumaroyl unit. The substituent positions of -OCH_3_ and -OH groups are opposite of **59**. The distinguishment in **59** and **60** is the same as that in **28** and **29**. They were all obtained from the plants *E*. *bodinieri*[17]. It is worth noting that one -O-CH_2_-O- group is connected with C-6 and C-7 of ring-A to get a furyl-ring in compound **63**.

Additionally, iso-formononetin-4'-*O*-β-D-glucopyranoside (**65**), amentoflavone (**66**), (+)-catechin (**67**), and (−)-epicatechin (**68**) were also isolated from *Elsholtzia*[11,19-22].

Compounds **6973** are linear furanocoumarins, in which **7073** were found in *E*. *densa* as new furanocoumarins [11,14,23]. **6972** exhibit a prenyl group or prenyl derivative in the C-5 position and a methoxy group in the C-8 position.

Only three lignanolides were reported in the genus. They are 3-hydroxyarctiin (**75**) and arctigenin (**76**) from *E*. *eriostachya*, together with saussurenoside (**77**) in *E*. *ianthina*[24,25].

### Terpenoids

Triterpenoids are other major constituents in this genus. The oleanane-type triterpenes (**8289**) were mainly isolated from the aerial part of *E*. *bodinieri*[26-29]. The glycosyl is linked to the C-28 (−COO-) of 23-hydroxyechinocystic acid by ester-bond in compounds **8789.** The C-3 position is attached a caffeoyl in compound **87**, and linked to an arabinosyl in **88** and **89**, respectively. Three ursane-type triterpenes including ursolic acid (**90**), corosolic acid (**91**) and 2α,3β,19α-trihydroxyurs-12-en-28-oic acid (**92**), are obtained from *E*. *rugulosa*, *E*. *ciliata* and *E*. *bodinieri*[17,30-32].

Two unusal 18,19-secoursane glycopyranosides, bodiniosides A (**93**) and B (**94**), were isolated from the whole plant of *E*. *bodinier*[17,27]. It was the first report that E-secoursane glycosides occurred in the Lamiaceae family. In addition, 2,3,19-trihydroxy urs-12-en-28-oic acid (**92**) and hypadienic acid (**95**) were also simultaneously obtained from the *E*. *bodinier*. Compounds **9395** could be derived from **92** in the biogenetic relationships [17].

Compounds **98102**, five diterpenoids, were isolated from *E*. *bodinier*[21,27,33,34]. Ludongnin 5 (**98**), a tetracyclic kaurane diterpenoid, is connected a γ-lactonic at the C6-C19 positions. **98** also has significant and extensive antibacterial effect [34,35]. Sandaracopimar-15-en-8β,12β-diol (**99**), a tricyclic pimarane diterpenoid, was isolated from *Elsholtzia* for the first time [21]. An abietane-type diterpenoid, (+)-hinokiol (**100**), is a minor diterpenoid occurring in plants. Its consuming inhibitive and deactive effects against *Staphylococcus aureus*, *Streptococcus*, *Escherichia coli*, and *Pseudomonas aeruginosa* attract the researchers [33]. It is the infrequence O-H…π stacking that was found in the packing of the crystal structure of **100** except for the existence of hydrogen bonding, when its molecular configuration and conformation were characterized by X-ray diffraction analysis [36]. Two hardwickiic acid glycopyranosides, 6-hydroxy-(−)-hardwickiic acid 2'-*O*-β-D-glucopyranosylbenzyl ester (**101**) and 6,7-dihydroxy-(−)-hardwickiic acid 2'-*O*-β-D-glucopyranosylbenzyl ester (**102**) were firstly reported in *E*. *bodinieri* as two novel clerodane diterpenoids [27].

Three eudesmane-type sesquiterpene glycopyranosides, dictamnoside G (**103**), 3β,5α,11,12,13-pentahydroxy-eudesm-4(15)-ene 3-*O*-β-D-apiofuranosyl-(1–4)-α-L-rhamnopyranosyl- (1–3)-β-D-glycopyranoside (**104**) and integrifoside A (**105**) were obtained from the root bark of *E*. *bodinieri*[37].

So far, only one monoterpenoid, 2,6-dimethyl-8-hydroxyl-2,6-octadienic acid-8-*O*-β-D-glucoside (**106**), was obtained from *E*. *bodinieri* as a new one [18].

### Others

Up to now, only three compounds (**107109**) containing nitrogen atoms were reported from the genus, all from *E*. *rugulosa*. Prunasin (**107**) and amygdalin (**108**) are cyanogenic glycosides [30,38,39]. This was the second report of cyanogenic glycosides in Lamiaceae plants. The first case was from an Australian plant *Clerodendrum grayi*. Since *Armeniacae semen* (apricot kernel) containing these compounds, it has been used for cough remedies in Europe and China. The result chemically supported the use of *E*. *rugulosa* for the treatment of colds and coughs in China. Three maltol glycosides, maltol 3-*O*-β-D-glucopyranoside (**110**), maltol 6'-*O*-β-D-apiofuranosyl-β-D-glucopyranoside (**111**) and maltol 6'-*O*-(5-*O*-p-coumaroyl)-β-D-apiofuranosyl-β-D-glucopyranoside (**112**) were isolated from *E*. *rugulosa*[38]. Here, **112** linked with a cinnamoyl can also be classified in the C6-C3 group. Besides, four phytosterols, **113116**, were reported from the genus [11,20-22,25-27,30-32,39,40], and a stilbene's hydroxylated derivative, *trans*-3,4,3',5'-tetrahydroxy-4^′^-methyl-stilbene 4-*O*-β-D-xylopyranosyl-(1→6)-β-D-glucopyranoside (**130**), was isolated from the root bark of *E*. *bodinieri*, as a new compound [33].

### Volatile chemical constituents

The plants *Elsholtzia* are aromatic herbs in general, as they possess plentiful volatile oils. The oils have been developed and utilized as medicines, food and the source of honey manufacture [1]. Many phytochemistry and pharmacology scientists are interested in the volatile constituents and its biological activities. The latest paper reported that the volatile constituents exert strong inhibition against central nervous system and take on considerate analgesic effect [41]. It also shows antibacterial effects [42-44].

A total of 572 volatile constituents were identified from the 21 species of *Elsholtzia* by hydro-distillation and gas chromatographic-mass spectrometry (GC-MS). Among them, α-pinene, β-pinene, acetophenone, caryophylene oxide, carvacrol, benzaldehyde, β-caryophyllene, 1,8-cineole, α-phellandrene, and α-terpineol widely exist. Especially, α-pinene and β-pinene are most significant two, which were detected and identified in 15 species in the genus [45-66]. Acetophenone, caryophylene oxide, carvacrol, benzaldehyde, β-caryophyllene, α-phellandrene, and α-terpineol are also major examples. It is a remarkable matter that the plant source, growing environment, harvesting time, extraction methods, and analysis methods of the study plants play important impact on the sorts and contents of some volatile components [45,67-70]. For instance, the content of α-pinene in *E*. *blanda* was up to 4.84% in Yunnan province, and decreased to 1.43% in Sichuan province, China [46,47]. A paper illustrated that the content and sort of volatile components from *E*. *stauntonii* were obviously contrasted with different extraction methods, respectively [67,69]. The researches foucing on the volatile components from *E*. *splendens*, *E*. *bodinieri*, *E*. *stauntonii*, and *E*. *ciliate* are much more than on other species [45,51,52,55,56,61-63,67-78]. The identified volatile components from the genus are shown in Additional file 4: Table S3, associated with the corresponding plant sources.

### Pharmacological activities

Pharmacological investigations on the extracts and pure compounds from *Elsholtzia* cover the activities of antiviral, antibacterial, anti-inflammatory, anti-oxidant, and myocardial ischemia protection, as well as other activities. Researchers are increasingly concerning on the pharmacological activities of the genus.

### Antiviral activity

Apigenin (**12**), apiin (**15**), luteolin (**16**), galuteolin (**20**), luteolin 3'-glucuronyl, methyl ester (**30**), and the ethyl acetate extract of *E*. *rugulosa* were reported to exhibit remarkable inhibition against the neuraminidases (Nas) from three typical influenza viruses A/PR/8/34 (H1N1), A/Jinan/15/90 (H3N2) and B/Jiangsu/10/2003. They inhibited influenza NAs at the different half maximal inhibitory concentration (IC_50_) values ranging from 7.81 μg/mL to 28.49 μg/mL. Especially, **12** and **16** exhibite significant effect against H3N2, with its IC_50_ values of 1.43 and 2.06 μg/mL, respectively. And the antiviral ability of **12** is 3 times higher than positive control, ribavirin. **16** has a similar capacity of antiviral activity with **12**[79]. Many *Elsholtzia* species, such as *E*. *bodinieri* and *E*. *blanda* contain rich luteolin (**16**) and its derivatives (**1623**) [80-82]. Its high content, such as up to 16.0 mg/g in the leaves of *E*. *blanda*, provide a convenient for the development of antiviral activity [80].

Essential oil from *E*. *densa* showed a significant inhibitory properties against Asia influenza virus A and Orphan virus *in vitro*. And it can postpone the symptom appearance by 72–96 h after being infected by virus *in vivo*. Also, it showed inhibition against H3N2 subtype of influenza A virus, and exhibited remarkable therapeutic effect on mouse pulmonic induced by influenza virus when the mouse was administered with the essential oil (100 mg/kg) [41].

### Antibacterial activity

Luteolin (**16**), quercetin (**33**) and ludongnin 5 (**98**) were isolated from the roots of *E*. *bodinieri*[9,21,26,34]. An antimicrobial assays indicated that these compounds had the inhibitory and bactericidal activities against *S*. *aureus*, *Bacillus subtilis* and *E*. *coli* in varying degrees. The minimal inhibitory concentrations (MIC) of 98 are 5, 10 and 80 μg/mL, respecitvely. The MIC values of 16 against *E*. *coli* and *S*. *aureus* 50 and 40 μg/mL and of 33 against *S*. *aureus* and *B*. *subtilis* with its MIC values of 60 and 90 μg/mL, respectively [34].

The ethanol extracts of *E*. *blanda* and *E*. *rugulosa* exhibited remarkable inhibitory activity against methicillin-resistant *S*. *aureus*, with its MIC values of 1.32 and 1.43 mg/mL, respectively [83].

Besides, some *Elsholtzia* essential oils also showed antimicrobial activity against bacterias, *i*.*e*. *E*. *coli*, *Shigeitn flexneri, S*. *epidermidis*, *beta Streptococcus*, *Bacterium paratyphosum B*, *B*. *typhi murium*, *B*. *dysenteriae*, *B*. *diphtheriae*, *B*. *meningitidis purulentae*, *B*. *proteus*, *Allthrax bacillus*, and *Neisseria intracellularis*[41].

Essential oils from *E*. *splendens* are inhibitory against *S*. *aureus*, *P*. *acnes* and *S*. *epidermidis*. Its MIC against *P*. *acnes* was 0.31 μL/mL. As we known, *P*. *acnes* and *S*. *epidermidis* are involved in the formation acne, thus the inhibition against the two bacteria supports the considerable potential of the *E*. *splendens* essential oil for the treatment of acne [42]. It was also reported that volatile components from *E*. *ciliata* and *E*. *rugulosa* inhibit common bacteria, such as *S*. *aureus*, *P*. *aeruginosa*, *B*. *enteritidis*, *B*. *subtilis*, *Proteus vularis*, *Shigella dysenteriae*, and *E*. *coli*[43,44].

### Anti-inflammatory

The 75% ethanol extract of the aerial part of *E*. *splendens* can significantly inhibit acute inflammation (mouse ear edema by croton oil-inducing) and subchronic inflammation (ear edema by phorbol ester inducing). *E*. *splendens* significantly inhibited PGE2 production by pre-induced cyclooxygenase-2 of lipopolysaccharide-treated RAW 264.7 cells. It was thus believed that inhibition against cyclooxygenase-2 is probably one of the function mechanisms [84].

On chemical view, luteolin (**16**), a widely contained flavone with many hydroxyl substitutions, is a bioactive constituent in *Elsholtzia* plants for anti-inflammatory activity. It can inhibit the production of NO and generation of other inflammatory cytokine, such as TNF-α, IL-1β, IL-6, NF-κB, etc. [41]. Therefore, rich production of hydroxylated flavone and/or its derivatives is one of reasons to some extend to explain the anti-inflammatory action for *Elsholtzia* plants.

### Anti-oxidant activity

The extracts of the aerial parts of *E*. *rugulosa* and *E*. *bodineri* displayed significant anti-oxidant activity in a radical-scavenged assay, which perhaps can elucidate why *E*. *rugulosa* has an anti-aging effect [85,86]. The flower of *E*. *rugulosa* is rich in flavonoids with its content up to 0.2352 mg/mL, and the flavonoids significantly scavenge the OH^-^ and O_2_^-^ ions, with its scavenging rate 30.8 and 40.5%, respectively [87]. The extract and extract-loaded nanoparticles of the flower of *E*. *splendens* showed a concentration-dependent manner in DPPH radical scavenging assay. *E*. *splendens* was also found to activate the antioxidant defense system against 7,12-dimethyl-benz(a)anthracene (DMBA)-induced oxidative stress and reduce several biomarkers of oxidative stress such as thio-barbituric acid reactive substance, protein carbonyls, serum 8-hydroxy-20-deoxyguanosine, and ovary CHO-K1 cells aging [88-92]. The maxium LPO inhibition ratio of the flavonoid extracts of *E*. *blanda* was 70.8% with the IC_50_ 0.23 mg/L. And the inhibition of LPO was induced by OH free radical [93]. The genus *Elsholtzia* owns rich polyphenols, thus having radical scavenging effects [94,95].

### Myocardial ischemia protection

The total flavones from *E*. *blanda* (TFEB) could improve the recovery of myocardial function, and keep heart from ischemic damage due to coronary occlusion in Beagle dogs. The effect was achieved by the inhibition of serum creatine kinase-MB (CK-MB) and malondialdehyde (MDA), together with by the lowing of mean arterial pressure (MAP), coronary vascular resistance (CVR), etc. [96]. The TFEB not only could reduce infarct size during acute myocardial infarction (AMI) by inhibiting myocardial apoptosis through modulation of Bcl-2 family [97], but also might decrease the myocardial ischemia and ‘Xiongbi Symtom’ [98]. Luteolin 7-*O*-β-D-glucopyranoside (**15**) could protect cultured neonatal rat cardiomyocytes from oxidative damage obviously, and the beneficial effects may be related to its anti-oxidant properties and reduction of intracellular calcium overload [99].

### Other activities

Besides the summarized functions above, the constituents or extracts from *Elshozia* plants have also some other activities. It is notable that apigenin (**12**) and luteolin (**16**) from *E*. *rugulosa* displayed protecting effects against the Alzheimer's disease (AD) in cell models. **12** protects rat cerebral microvascular endothelial cells (CMECs) against amyloid-β peptide 25–35 (Aβ_25–35_) -induced toxicity. Endothelial cells of cerebral capillaries forming the blood–brain barrier play an important role in the pathogenesis and therapy of AD. Aβ _25–35_ showed toxicity on CMECs, and breaked the barrier integrity and function [100]. Copper can trigger the neurotoxicity in amyloid precursor protein Swedish (APPsw) overexpressing cells, which exacerbated the amyloid-β (Aβ) neurotoxicity and can be taken as a model of AD. Luteolin (**16**) treatment exerted neuroprotection through mechanisms that decrease amyloid-β precursor protein (AβPP) expression, lower Aβ secretion, regulate the redox imbalance, preserve mitochondrial function, and depress caspase family-related apoptosis [101]. *E*. *splendens* exhibited significant analgesic activity against mouse acetic acid-induced writhing. And the inhibition is up to 50% at 400 mg/kg [84].

A study indicated that *E*. *splendens* could obviously relieve symptoms of premenstrual syndrome. And the scores of depression and anxiety and the premenstrual instability decreased significantly [102]. The extracts of *E*. *splendens* had the potential of inducing structural aberration of chromosome. The ethanolic extracts of *E*. *splendens* have been found for reducing blood lipid by lowing low-density lipoprotein (LDL)-cholesterol [103,104]. And ‘Ciwujia Xiangru Decoction’, one of Chinese Traditional Compound Medicines consisting of *Acanthopanax senticosus* and *E*. *splendens*, has been investigated the mentioned effect and used in clinic [105]. The extracts of *E*. *splendens* and *E*. *stauntonii* had fumigant toxicity, and may be potential fumigants for integrated pest management programs of stored-grain insect [106,107]. Additionally, *E*. *bodinieri* extracts exhibited certain effect on depressing blood-lipid by reducing the level of total cholesterol of rats [108].

## Conclusion remarks

The review paper summarized a total of 144 compounds and abundant volatile components that were reported from the genus *Elsholtzia*, with 117 references cited. We noted that *Elsholtzia* has an extensive distribution, diverse biological and pharmacological activities of pure compounds, extracts and volatile components described. Previous phytochemical researches on the genus revealed the extensive presence of flavone, coumarin, terpenoid, and other compound types, together with prolific essential oils. The pharmacological activities of volatile constituents mainly were regarded on antioxidant, antiviral and antibacterial activities.

From the review, it can be seen that phytochemical investigations mainly focus on 10 *Elsholtzia* species, *E*. *blanda*, *E*. *bodinieri*, *E*. *ciliata*, *E*. *cristata*, *E*. *densa*, *E*. *eriostachya*, *E*. *ianthina*, *E*. *rugulosa*, *E*. *splendens*, and *E*. *stauntonil*. And the volatile constituents’ analyses primarily concentrated on 20 species. However, related chemical and biological toward other *Elsholtzia* species, including *E*. *kachinensis*, *E*. *capituligera*, *E*. *cephalantha*, *E*. *cyprianii*, *E*. *eriocalyx*, *E*. *flava*, *E*. *glabra*, *E*. *heterophylla*, *E*. *hunannensis*, *E*. *kachinensis*, *E*. *luteola*, *E*. *ochroleuca*, *E*. *oldhamii*, *E*. *penduliflora*, *E*. *pilosa*, *E*. *pygmaea*, *E*. *saxatilis*, *E*. *souliei*, *E*. *stachyodes*, and *E*. *winitiana*, are still blank. So, plenty of further studies are necessary in order to illustrate the chemo-diversity and to make full use of the biological significance of the compounds and extracts of *Elsholtzia,* especially the antiviral and anti-inflammatory activities. The authors wish the review can provide a valuable data for explorations and advanced researches of *Elsholtzia* species.

## Competing interests

The authors declare that they have no competing interests.

## Authors’ contributions

GZ, LZ, WX, and SG have been involved in preparing the manuscript. LW, JR and CR participated in the discussion of views in the paper. All authors have read and approved the final manuscript.

## Supplementary Material

Additional file 1 **Table S1.** The list of *Elsholtzia* species [1,2]. Click here for file

Additional file 2 **Table S2.** The name, plant source of compounds 1–144 from *Elsholtzia*[9-33,35,38-42,106,109-114]. Click here for file

Additional file 3 **Figure S1.** The structures of compounds 1–132 from *Elsholtzia.* Click here for file

Additional file 4 **Table S3.** The volatile chemical components from *Elsholtzia*[41,43,44,47-49,51-79,106,114-118]. Click here for file
