# Supplementary material for: Elsholtzia: phytochemistry and biological activities
Source: Chem Cent J. 2012 Dec 5;6:147. doi: 10.1186/1752-153X-6-147 (PMC3536681; doi:10.1186/1752-153X-6-147)
Supplement: Additional file 1 — Table S1. The list of Elsholtzia species [1,2]. [file 1752-153X-6-147-S1.doc]

**Additional file 1: Table S1**. The list of *Elsholtzia* species [1,2]

| **No.** | **Species** | **Chinese** **name** | **Distribution** | **Altitude** **(m)** |
| --- | --- | --- | --- | --- |
| 1 | *E*. *argyi* | Zi Hua Xiang Ru, Ya Shua Cao, Jing Jie Cao, Ye Bo He, Chou Cao, Jia Zi Su, Tu Jing Jie, and Jin Ji Cao. | Guizhou, Zhejiang, Jiangsu, Anhui, Fujian, Jiangxi, Guangdong, Guangxi, Hunan, Hubei, Guizhou, Sichuan (China); and Japan, Vietnam (cultivated). | 200–1200 |
| 2 | *E*. *blanda* | Si Fang Hao, Yan He He Luo, Hei Tou Cao, Si Heng Hao, Ji Gan San, Tie Sao Ba, Ye Bo He, Ye Su, Man Ba, and Jing Jie. | Yunnan, Guizhou, Guangxi (China), Nepal, Sikkim, Bhutan | 800–2500 |
|  |  |  | Myanmar, Thailand, Laos, India, Vietnam, and Indonesia. |  |
| 3 | *E*. *bodinieri* | Dong Zi Su, Tie Xian Xia Ku Cao, Xiao Song Mao Cha, Ban Bian Hong Hua, Ya Shua Cao, Xiao Xiang Cha, Xiao Shan Cha, Xiao Xiang Ru, Yun Song Cha, Feng Wei Cha, Xiu Shan Cha, Xiang Su Cha, Xiao Ye Cha, Ye Shan Cha, Shan Cha Ye, Shan Cha, and Ya Zi Cao. | Yunnan and Guizhou (China). | 1200–3000 |
| 4 | *E*. *capituligera* | Tou Hua Xiang Ru. | Yunnan, Sichuan, and Tibet (China). | 2000–3000 |
| 5 | *E*. *cephalantha* | Xiao Tou Hua Xiang Ru. | Sichuan (China). | 3200–4100 |
| 6 | *E*. *ciliate* | Xiang Ru, Yu Xiang Cao, Xiao Ye Su Zi, Xiao Jing Jie, Shan Su Zi, and La La Cao. | All provinces except qinghai and xinjiang in China; Cambodia, India, Japan, Laos, Malaysia, Mongolia, Myanmar, Russia, Thailand, Vietnam, introduced in Europe and North of America. | 0–3400 |
| 7 | *E*. *communis* | Ji Long Cao and Xian Luo Xiang Cai | Yunnan (cultivated) (China), Myanmar, Thailand | 800–1000 |
| 8 | *E*. *cyprianii* | Mao Wei Xiang Ru, Man Shan Xiang, Gou Wei Ba Cao, Ye Cao Xiang, Ye Huo Su, and Ye Su Ma. | Yunnan, Guizhou, Sichuan, Henan, Anhui, Hubei, Hunan, Guangxi, and Shaanxi (China). | 400–2900 |
| 9 | *E*. *densa* (*E*. *calycocarpa*) | Mi Hua Xiang Ru, Chou Xiang Ru, Xi Xi Bian, Hai La Cao, and Ye Zi Su. | Yunnan, Sichuan, Hebei, Liaoning, Qinghai, Shaanxi, Xinjiang, Gansu, Shanxi (China); Afghanistan, India, Sikkim, Nepal, Pakistan, Taigikistan. | 1000–4100 |
| 10 | *E*. *eriocalyx* | Mao E Xiang Ru. | Yunnan, Sichuan (China). | 2700–3400 |
| 11 | *E*. *eriostachya* | Mao Sui Xiang Ru. | Yunnan, Sichuan, Tibet, Gansu (China); Nepal, Sikkim, and India. | 3500–4100 |
| 12 | *E*. *feddei* | Gao Yuan Xiang Ru. | Gansu, Hebei, Qinghai, Shaanxi, Shanxi, Yunnan, Sichuan, and Tibet (China). | 500–3200 |
| 13 | *E*. *flava* | Huang Hua Xiang Ru, Da Ye Xiang Zhi Ma, Da Ye Xiang Ru, Xiu Xian Guo, Da Ye Ba Ai. | Yunnan, Sichuan, Tibet, Hubei, Guizhou, Zhejiang (China); Sikkim, Nepal, and India. | 1000–2900 |
| 14 | *E*. *fruticosa* | Ji Gu Chai, Shou Gou Huan Yang Cao, Shuang Ling Cao, Xiang Zhi Ma Ye, Lao Ma Ma Cao, Shan Ye Bao Zi, and Sao Di Cha. | Yunnan, Sichuan, Tibet, Gansu, Hubei, Guizhou, Guangxi (China); Sikkim, Nepal, Bhutan, and India. | 1200–3800 |
| 15 | *E*. *glabra* | Guang Xiang Ru. | Yunnan and Sichuan (China). | 1900–2400 |
| 16 | *E*. *heterophylla* | Yi Ye Xiang Ru. | Yunnan (China) and Myanmar. | 1200–2400 |
| 17 | *E*. *hunannensis* | Hu Nan Xiang Ru. | Hunan, Anhui, Guizhou, Hubei, Jiangxi (China). | 200–2500 |
| 18 | *E*. *kachinensis* | Shui Xiang Ru, Zhu Cai Cao, An Nan Mu, and Shui Bao. | Yunnan, Guizhou, Sichuan, Tibet, Jiangxi, Hunan, Hubei, Guangdong, Guangxi (China); and Myanmar. | 1200–2800 |
| 19 | *E*. *luteola* | Dan Huang Xiang Ru. | Yunnan, Sichuan (China). | 2200–3600 |
| 20 | *E*. *myosurus* | Shu Wei Xiang Ru, Mi Hua Xiang Ru, and Da Xiang Hua Ke. | Yunnan and Sichuan (China). | 2600–3000 |
| 21 | *E*. *ochroleuca* | Huang Bai Xiang Ru. | Yunnan and Sichuan (China). | 1600–2600 |
| 22 | *E*. *oldhamii* | Tai Wan Xiang Ru. | Taiwan (China). |  |
| 23 | *E*. *penduliflora* | Da Huang Yao, Ye Su Zi Ke, Ye Zhi Ma, and Huang Yao. | Yunnan (China). | 1100–2400 |
| 24 | *E*. *pilosa* | Chang Mao Xiang Ru, Da Ru. | Yunnan, Sichuan, Guizhou (China);, Sikkim, Nepal, India, Vietnam, and Myanmar. | 1100–3200 |
| 25 | *E*. *pygmaea* | Ai Xiang Ru. | Lijiang (China). |  |
| 26 | *E*. *rugulosa* | Xiao Xiang Zhi Ma Ye, Gou Wei Ba Xiang, Xiang Zhi Ma Hao, Ban Bian Xiang, Chou Xiang Ru, Qing Niu Teng, Hao Ba Ba Ke, Xiang Zhi Ma, Xiang Su Cao, Di Tan Xiang, Xiao Xiao Su, Xiao Shan Su, Ye Xiang Su, Sao Ba Cha, Bai Bei Hao, Xiao Zi Su, Ye Ba Hao, Ye Ba Cao, Ba Zi Cao, Xiao Tie Su, La You Ma, Gou Ba Zi, Cao Ba Zi, Song Hua, Ye Ba Zi, Ye Su Zi, Tie Su Su, Tie Su Ke, and Ye Su. | Yunnan, Sichuan, Guizhou, and Guangxi (China). | 1300–2800 |
| 27 | *E*. *saxatilis* | Yan Sheng Xiang Ru. | Heilongjiang, Jilin, Liaoning, Shandong (China); Japan, Korea, and Russia (Siberia). |  |
| 28 | *E*. *souliei* | Chuan Dian Xiang Ru. | Yunnan and Sichuan (China). | 2800–3300 |
| 29 | *E*. *splendens* | Hai zhou Xiang Ru. | Liaoning, Hebei, Shandong, Jiangsu, Jiangxi, Zhejiang, Henan, Hubei, Guangdong, (China); and Korea. | 200–300 |
| 30 | *E*. *stachyodes* | Sui Zhuang Xiang Ru. | Yunnan, Shaanxi, Hubei, Sichuan, Guizhou, Guangxi, Guangdong, Zhejiang, Anhui (China); Nepal, India, and Myanmar. | 800–2800 |
| 31 | *E*. *stauntonii* | Mu Xiang Ru, Xiang Jing Jie, Shan Jing Jie, Chou Jing Jie, Ye Jing Jie, and Zi Jing Jie. | Hebei, Shanxi, Shaanxi, Gansu, and Henan (China). | 700–1600 |
| 32 | *E*. *strobilifera* | Qiu Sui Xiang Ru, Chou Su Ma, and Ye Su Ma. | Yunnan, Sichuan, Tibet, Taiwan (China); Nepal, and India. | 2300–3700 |
| 33 | *E*. *winitiana* | Bai Xiang Ru, Xiang Ru, Ma Yong Ya, and Si Fang Hao. | Yunnan and Guagnxi (China). | 600–2200 |
