# Supplementary material for: Elsholtzia: phytochemistry and biological activities
Source: Chem Cent J. 2012 Dec 5;6:147. doi: 10.1186/1752-153X-6-147 (PMC3536681; doi:10.1186/1752-153X-6-147)
Supplement: Additional file 2 — Table S2. The name, plant source of compounds 1–144 from Elsholtzia[9-33,35,38-42,106,109-114]. [file 1752-153X-6-147-S2.doc]

***Additional file 2: Table S2.*** *The name, plant source of compounds 1–144 from Elsholtzia*

| **No.** | **Name** | **Source** | **Reference** |
| --- | --- | --- | --- |
| **Flavonoids** | | | |
| **1** | 5-hydroxy-6, 7-dimethoxyflavone | *E*. *splendens* | [9] |
|  |  | *E*. *stauntonii* | [14] |
|  |  | *E*. *blanda* | [17,109] |
|  |  | *E*. *ciliata* | [32,110] |
| **2** | 5, 6-dihydroxy-7, 8-dimethoxyflavone | *E*. *splendens* | [14] |
| **3** | 5-hydroxy-7, 8-dimethoxyflavone | *E*. *splendens* | [14] |
|  |  | *E*. *blanda* | [109] |
|  |  | *E*. *ciliata* | [32,110] |
| **4** | 5, 7-dihydroxyflavone | *E*. *bodinieri* | [10,27,28] |
| **5** | negletein | *E*. *ciliata* | [33] |
| **6** | 5, 6-dihydroxy-7-methoxyflavone −6-*O*- [α-L-rhanmopyranosyl (1→2) -β-D-fucopyranosyl] glycoside | *E*. *blanda* | [16,17] |
| **7** | 4', 5-dihydroxy-7-methoxyflavone | *E*. *rugulosa* | [31] |
| **8** | 5-hydroxy-4', 6, 7-trimethoxyflavone | *E*. *rugulosa* | [31] |
| **9** | acacetin | *E*. *densa* | [111] |
|  |  | *E*. *bodinieri* | [22,27] |
|  |  | *E*. *ciliata* | [32,110] |
| **10** | 5, 7-dihydroxy-4'-methoxyflavone-7-*O*-rutinoside | *E*. *densa* | [24,111] |
|  |  | *E*. *cristata* | [112] |
|  |  | *E*. *eriostachya* | [113] |
| **11** | acacetin 7-*O*-β-D-glucopyranoside | *E*. *ciliata* | [32] |
|  |  | *E*. *rugulosa* | [40] |
| **12** | apigenin | *E*. *bodinieri* | [10] |
|  |  | *E*. *ciliata* | [33] |
|  |  | *E*. *rugulosa* | [11,31,40-42] |
|  |  | *E*. *cristata* | [112] |
| **13** | apigenin 4'-*O*-α-D-glucopyranoside | *E*. *rugulosa* | [11] |
| **14** | apigenin 7-*O*-β-D-glucopyranoside | *E*. *bodinieri* | [10] |
|  |  | *E*. *rugulosa* | [11,40] |
|  |  | *E*. *cristata* | [112] |
| **15** | apiin | *E*. *rugulosa* | [42] |
| **16** | luteolin | *E*. *bodinieri* | [10,27,35] |
|  |  | *E*. *rugulosa* | [11,39,40,42] |
|  |  | *E*. *ciliata* | [33] |
|  |  | *E*. *blanda* | [109] |
| **17** | luteolin 5-*O*-β-D-glucopyranoside | *E*. *blanda* | [109] |
|  |  | *E*. *eriostachya* | [25,113] |
| **18** | luteolin 3'-*O*-β-D-glucopyranoside | *E*. *blanda* | [114] |
| **19** | luteolin 3'-*O*-β-D- glucuronide | *E*. *rugulosa* | [39] |
| **20** | glucoluteolin | *E*. *bodinieri* | [10,19,27] |
|  |  | *E*. *eriostachya* | [31,113] |
|  |  | *E*. *rugulosa* | [11,39,40,42] |
|  |  | *E*. *blanda* | [109,114] |
|  |  | *E*. *cristata* | [112] |
| **21** | luteolin 7-*O*-β-D-galactoside | *E*. *blanda* | [114] |
| **22** | 5, 7-dimethoxyl-4'-hydroxyflavone | *E*. *stauntonii* | [12] |
| **23** | 5, 7, 3', 6'-tetrahydroxy-8, 2'-dimethoxyflavone | *E*. *stauntonii* | [13] |
| **24** | 5, 6-dihydroxy-3', 4', 7, 8-tetramethoxyflavone | *E*. *rugulosa* | [31] |
| **25** | 5-hydroxy-7, 5'-dimethoxy-6, 8-dimethyl-3', 4'-methylenedioxy flavone | *E*. *stauntonii* | [14] |
| **26** | tricin | *E*. *bodinieri* | [22] |
| **27** | luteolin 7-*O*- (6"-acetyl) -β-D-glucopyranoside | *E*. *rugulosa* | [40] |
| **28** | luteolin 7-*O*- (6″-feruloyl) -β-D-glucopyranoside | *E*. *bodinieri* | [10] |
| **29** | luteolin 7-*O*- [6"- (3"'-hydroxy-4"'-methoxy cinnamoyl)] -β-D- glucopyranoside | *E*. *bodinieri* | [10] |
| **30** | luteolin 3'-*O*-β-D-glucuronide-6''-methylester | *E*. *rugulosa* | [11,42] |
| **31** | kumatakenin | *E*. *ciliata* | [21] |
| **32** | 7, 4'-dimethoxy kaempferol | *E*. *rugulosa* | [11,41] |
| **33** | quercetin | *E*. *rugulosa* | [31] |
|  |  | *E*. *bodinieri* | [22,35] |
| **34** | kaempferol | *E*. *rugulosa* | [11,31] |
|  |  | *E*. *bodinieri* | [20] |
|  |  | *E*. *ianthina* | [26] |
| **35** | morin 7-*O*-β-D-glucopyranoside | *E*. *eriostachya* | [25,113] |
| **36** | kaempferol 3-*O*-β-D-glucopyranoside | *E*. *densa* | [24,111] |
| **37** | isoatragalin | *E*. *blanda* | [114] |
| **38** | kaempferol 3-*O*-rutinnoside | *E*. *bodinieri* | [19] |
| **39** | quercetin 3-*O*-β-D-glucopyranoside | *E*. *densa* | [24,111] |
|  |  | *E*. *rugulosa* | [39] |
| **40** | rutin | *E*. *bodinieri* | [19] |
| **41** | hyperoside | *E*. *eriostachya* | [25,113] |
| **42** | quercetin 3-*O*-β-D-gal (6→1) -α-L-rha | *E*. *stauntoniii* | [12] |
| **43** | isorhartmetin 3-*O*-rutinoside | *E*. *stauntonii* | [12] |
| **44** | quercetin 3-*O*-β-D-glucuronide-6''-methylester | *E*. *rugulosa* | [11] |
| **45** | 5, 7, 3', 4'-tetrahydroxy-8-prenylflavone | *E*. *rugulosa* | [11] |
| **46** | 5, 7, 3', 4'-tetrahydroxy-5'-C-prenylflavone 7-*O*-β-D-glucoside | *E*. *rugulosa* | [11] |
| **47** | muxiangrine III | *E*. *stauntonii* | [12,13] |
| **48** | 5-hydroxy-7-methoxy-8-methyl-3', 4'-methylenedioxy-5'- (3-methyl-but-2-enyl) -3', 4'-methylenedioxy flavone | *E*. *stauntonii* | [14] |
| **49** | 3"-hydroxy-4", 5"-dimethoxyfuranoflavone | *E*. *densa* | [15] |
| **50** | 3", 4", 5"-trimethoxyfuranoflavone | *E*. *densa* | [15] |
| **51** | muxiangrine I | *E*. *stauntonii* | [12,13] |
| **52** | muxiangrine II | *E*. *stauntonii* | [12,13] |
| **53** | sifanghaoine I | *E*. *blanda* | [16,17] |
| **54** | sifanghaoine II | *E*. *blanda* | [16,17] |
| **55** | sifanghaoine III | *E*. *blanda* | [16,17] |
| **56** | eriodictyol | *E*. *bodinieri* | [10] |
| **57** | 5-hydroxy-6-methylflavanone 7-*O*-α-D-galacoside | *E*. *ciliata* | [32,110] |
| **58** | eridodictyol 7-*O*-β-D-glucopyranoside | *E*. *bodinieri* | [19] |
| **59** | eriodictyol 7-*O*- (6"'-feruloyl) -β-D-glucopyranoside | *E*. *bodinieri* | [10] |
| **60** | eriodictyol 7-*O*- [6"- (3"'-hydroxy-4"'-methoxy cinnamoyl)] -β-D- glucopyranoside | *E*. *bodinieri* | [10] |
| **61** | isoskuranetin 7-*O*-β-D-neohesperidoside | *E*. *eriostachya* | [25,113] |
| **62** | 5-hydroxy-3'-methoxyflavanone-7-*O*-rutinoside | *E*. *densa* | [24,111] |
| **63** | 5, 2'-dimethoxy-6, 7-methylenedioxy flavanone | *E*. *blanda* | [16] |
| **64** | 5-hydroxy-7, 4'-dimethoxyflavanonol | *E*. *stauntonii* | [14] |
|  |  | *E*. *ciliata* | [32,110] |
|  |  | *E*. *densa* | [107] |
| **65** | iso-formononetin 4'-*O*-β-D-glucopyranoside | *E*. *stauntonii* | [12] |
| **66** | amentoflavone | *E*. *bodinieri* | [23] |
| **67** | (+) –catechin | *E*. *bodinieri* | [20] |
|  |  | *E*. *ciliata* | [21] |
| **68** | epi-gallocatechin | *E*. *bodinieri* | [22] |
| **Phenylpropanoids** | | | |
| **69** |  | *E*. *stauntonii* | [12] |
| **70** | 5- (3"-methylbutyl) -8-methoxyfuranocoumarin | *E*. *stauntonii* | [12] |
|  |  | *E*. *densa* | [15,24] |
| **71** | 5- (3"-hydroxy-3-methylbutyl) -8-methoxyfuranocoumarin | *E*. *densa* | [15,24] |
| **72** | 5- (3", 3"-dimethylally) -8-methoxyfuranocoumarin | *E*. *densa* | [15,24] |
| **73** | 9-O-isopropyl-4-methoxypsoralen | *E*. *densa* | [106] |
| **74** | 6-hydroxy-5, 7-dimethoxycoumarin | *E*. *bodinieri* | [35] |
| **75** | 3-hydroxyarctiin | *E*. *eriostachya* | [25] |
| **76** | aretigenin | *E*. *eriostachya* | [25] |
| **77** | saussurenoside | *E*. *ianthina* | [26] |
| **78** | caffeic acid | *E*. *ciliata* | [21] |
|  |  | *E*. *bodinieri* | [35] |
|  |  | *E*. *rugulosa* | [39] |
| **79** | stearyl ferulate | *E*. *ciliata* | [21] |
| **80** | rosmarinic acid | *E*. *bodinieri* | [23] |
|  |  | *E*. *rugulosa* | [39] |
| **81** | 4-allyl-2, 6-dimethoxyphenol 1-*O*-β-D-glucoside | *E*. *bodinieri* | [35] |
| **Terpenes** | | | |
| **82** | oleanolic acid | *E*. *ianthina* | [26] |
|  |  | *E*. *bodinieri* | [27,28] |
|  |  | *E*. *rugulosa* | [31] |
| **83** | maslinic acid | *E*. *rugulosa* | [40] |
| **84** | hederagenin | *E*. *bodinieri* | [29] |
| **85** | dodecandral 3-*O*-β-D-xylopyranoside | *E*. *bodinieri* | [29] |
| **86** | hederagenin 3-*O*-β-D-xylopyranoside | *E*. *bodinieri* | [29] |
| **87** | bodinierin C | *E*. *bodinieri* | [30] |
| **88** | ciwujianoside C | *E*. *bodinieri* | [30] |
| **89** | mazusaponin I | *E*. *bodinieri* | [30] |
| **90** | ursolic acid | *E*. *rugulosa* | [31] |
|  |  | *E*. *ciliata* | [32,33] |
| **91** | corosolic acid | *E*. *ciliata* | [33] |
|  |  | *E*. *rugulosa* | [40] |
| **92** | 2, 3, 19-trihydroxy urs-12-en-28-oic acid | *E*. *bodinieri* | [18] |
|  |  | *E*. *ciliata* | [33] |
| **93** | bodinioside A | *E*. *bodinieri* | [18] |
| **94** | bodinioside B | *E*. *bodinieri* | [18,28] |
| **95** | hypadienic acid | *E*. *bodinieri* | [18] |
| **96** | betulinic acid | *E*. *rugulosa* | [40,41] |
| **97** | friedelin | *E*. *blanda* | [16] |
| **98** | ludongnin 5 | *E*. *bodinieri* | [35] |
| **99** | sandaracopimar-15-en-8b, 12β-diol | *E*. *bodinieri* | [22] |
| **100** | (+) –hinokiol | *E*. *bodinieri* | [35] |
| **101** | 6-hydroxy- (−) -hardwickiic acid 2'-*O*-β-D-glucopyranosylbenzyl ester | *E*. *bodinieri* | [28] |
| **102** | 6, 7-dihydroxy- (−) -hardwickiic acid 2'-*O*-β-D-glucopyranosylbenzyl ester | *E*. *bodinieri* | [28] |
| **103** | dictamnoside G | *E*. *bodinieri* | [38] |
| **104** | 3β, 5α, 11, 12, 13-pentahydroxy-eudesm-4 (15) -ene 3-*O*-β-D- apiofuranosyl- (1→4) -α-L-rhamnopyranosyl- (1→3) -β-D- glucopyranoside | *E*. *bodinieri* | [38] |
| **105** | integrifoside A | *E*. *bodinieri* | [38] |
| **106** | bodinierin | *E*. *bodinieri* | [19] |
| **Others** | | | |
| **107** | prunasin | *E*. *rugulosa* | [39,40] |
| **108** | amygdalin | *E*. *rugulosa* | [39] |
| **109** | 1H-indole-3-carboxylic acid | *E*. *rugulosa* | [31] |
| **110** | maltol 3-*O*-β-D-glucoside | *E*. *rugulosa* | [39] |
| **111** | maltol 6'-*O*-β-D-apiofuranosyl-β-D-glucopyranoside | *E*. *rugulosa* | [39] |
| **112** | maltol 6'-*O*-(5-*O*-p-coumaroyl)- β-D-apiofuranosyl-β-D- glucopyranoside | *E*. *rugulosa* | [39] |
| **113** | ergosta-7-en-3β-ol | *E*. *rugulosa* | [31] |
| **114** | stigmasteol | *E*. *rugulosa* | [41] |
| **115** | β-sitosterol | *E*. *stautonii* | [12] |
|  |  | *E*. *bodinieri* | [22,27] |
|  |  | *E*. *ianthina* | [26] |
|  |  | *E*. *ciliata* | [32,33] |
|  |  | *E*. *rugulosa* | [31,40,41] |
| **116** | dacuosterol | *E*. *bodinieri* | [23,27,28] |
|  |  | *E*. *rugulosa* | [31] |
|  |  | *E*. *ciliata* | [21,32,33] |
| **117** | vanillic acid | *E*. *bodinieri* | [35] |
| **118** | 2-hydroxymethyl-5-methoxyphenyl-*O*-β-D-glucopyranoside | *E*. *bodinieri* | [23] |
| **119** | bodinierine A | *E*. *bodinieri* | [23] |
| **120** | 4-hydroxy-2, 6-dimethoxyphenol 1-*O*-β-D-glucopyranoside | *E*. *bodinieri* | [35] |
| **121** | glucosyringic acid | *E*. *blanda* | [16] |
| **122** | gentisic acid 5-*O*-β-D-glucopyranoside | *E*. *eriostachya* | [25] |
| **123** | gentisic acid 5-*O*-β-D-glucoside | *E*. *eriostachya* | [25] |
| **124** | 4-hydroxy-3-methoxystyrene | *E*. *blanda* | [16] |
| **125** | vanillin | *E*. *bodinieri* | [20] |
| **126** | gallic acid | *E*. *bodinieri* | [20] |
| **127** | 5, 6-dihydro-6-styry-2-pyrone | *E*. *blanda* | [16] |
| **128** | benzyl alcoholβ-D-glucopyranoside | *E*. *rugulosa* | [39,40] |
| **129** | bodinieriside C | *E*. *bodinieri* | [20] |
| **130** | trans-3, 4, 3', 5'-tetrahydroxy-4′-methyl-stilbene 4-*O*-β-D-xylopyranosyl- (1→6) -β-D-glucopyranoside | *E*. *bodinieri* | [35] |
| **131** | tuberonic acid β-D-glucoside | *E*. *rugulosa* | [39] |
| **132** | α-L-n-butylsorboside | *E*. *stauntonii* | [12] |
| **133** | succinic acid | *E*. *densa* | [24] |
| **134** | sorbic acid | *E*. *rugulosa* | [41] |
| **135** | linolenic acid | *E*. *ciliata* | [32] |
| **136** | linoleic acid | *E*. *ciliata* | [32] |
| **137** | palmitic acid | *E*. *ciliata* | [32] |
| **138** | hexacosanol | *E*. *ianthina* | [26] |
| **139** | 6-methyl dotracontane | *E*. *stauntonii* | [12] |
| **140** | hexatriacontane | *E*. *rugulosa* | [41] |
| **141** | eicosane | *E*. *rugulosa* | [41] |
| **142** | n-nonacosane | *E*. *densa* | [24] |
| **143** | 6-methyl tritriacontane | *E*. *ciliata* | [32] |
| **144** | 13-cyclohexyl-hexoacosane | *E*. *ciliata* | [32] |
