# Supplementary material for: Elsholtzia: phytochemistry and biological activities
Source: Chem Cent J. 2012 Dec 5;6:147. doi: 10.1186/1752-153X-6-147 (PMC3536681; doi:10.1186/1752-153X-6-147)
Supplement: Additional file 3 — Figure S1. The structures of compounds 1–132 from Elsholtzia. [file 1752-153X-6-147-S3.doc]

| No. | **R_1_** | **R_2_** | **R_3_** | **R_4_** | **R_5_** | **R_6_** |
| --- | --- | --- | --- | --- | --- | --- |
| **1** | OH | OCH_3_ | OCH_3_ | H | H | H |
| **2** | OH | OH | OCH_3_ | OCH_3_ | H | H |
| **3** | OH | H | OCH_3_ | OCH_3_ | H | H |
| **4** | OH | H | OH | H | H | H |
| **5** | OH | OH | OCH_3_ | H | H | H |
| **6** | OH | *O*-β-D-fuc (2-1) α-L-rha | OCH_3_ | H | H | H |
| **7** | OH | H | OCH_3_ | H | H | OH |
| **8** | OH | OCH_3_ | OCH_3_ | H | H | OCH_3_ |
| **9** | OH | H | OH | H | H | OCH_3_ |
| **10** | OH | H | *O*-β-D-glc (6-1) α-L-rha | H | H | OCH_3_ |
| **11** | OH | H | *O*-β-D-glc | H | H | OCH_3_ |
| **12** | OH | H | OH | H | H | OCH_3_ |
| **13** | OH | H | OH | H | H | *O*-α-D-gal |
| **14** | OH | H | *O*-β-D-glc | H | H | OH |
| **15** | OH | H | *O*-β-D-glc (2-1) β-D-api | H | H | OCH_3_ |
| **16** | OH | H | OH | H | OH | OH |
| **17** | *O*-β-D-glc | H | OH | H | OH | OH |
| **18** | OH | H | OH | H | *O*-β-D-glc | OH |
| **19** | OH | H | OH | H | *O*-β-D-glcA | OH |
| **20** | OH | H | *O*-β-D-glc | H | OH | OH |
| **21** | OH | H | *O*-β-D-gal | H | OH | OH |
| **22** | OCH_3_ | H | OCH_3_ | H | H | OH |

| No. | **R_1_** | **R_2_** | **R_3_** | **R_4_** | **R_5_** |
| --- | --- | --- | --- | --- | --- |
| **31** | OCH_3_ | OCH_3_ | H | H | OH |
| **32** | OH | OCH_3_ | H | H | OCH_3_ |
| **33** | OH | OH | H | OH | OH |
| **34** | OH | OH | H | H | OH |
| **35** | OH | *O*-β-D-glc | OH | H | OH |
| **36** | *O*-β-D-glc | OH | H | H | OH |
| **37** | *O*-α-D-glc | OH | H | H | OH |
| **38** | *O*-β-D-glc (6-1) α-L-rha | OH | H | H | OH |
| **39** | *O*-β-D-glc | OH | H | OH | OH |
| **40** | *O*-β-D-glc (6-1) α-L-rha | OH | H | OH | OH |
| **41** | *O*-β-D-gal | OH | H | OH | OH |
| **42** | *O*-β-D-gal (6-1) α-L-rha | OH | H | OH | OH |
| **43** | *O*-β-D-glc (6-1) α-L-rha | OH | H | H | OH |

| No. | **R_1_** | **R_2_** | **R_3_** | **R_4_** | **R_5_** | **R_6_** |
| --- | --- | --- | --- | --- | --- | --- |
| **45** | H | OH | A | OH | OH | H |
| **46** | H | *O*-β-D-glc | H | OH | OH | A |
| **47** | CH_3_ | OCH_3_ | CH_3_ | OH | OH | A |
| **48** | H | OCH_3_ | CH_3_ | O-CH_2_-O | | A |

| No. | **R_1_** | **R_2_** | **R_3_** |
| --- | --- | --- | --- |
| **51** | CH_3_ | OCH_3_ | CH_3_ |
| **52** | CH_3_ | OCH_3_ | H |
| **53** | CH_3_ | OOCCH_3_ | CH_3_ |
| **54** | CH_3_ | COCH(CH_3_)CH_2_CH_3_ | CH_3_ |
| **55** | O-CH_2_-O | | CH_3_ |

| No. | **R_1_** | **R_2_** | **R_3_** | **R_4_** | **R_5_** | **R_6_** |
| --- | --- | --- | --- | --- | --- | --- |
| **56** | OH | H | OH | H | OH | OH |
| **57** | OH | CH_3_ | *O*-α-D-glc | H | H | H |
| **58** | OH | H | *O*-β-D-glc | H | OH | OH |
| **59** | OH | H | *O*-β-D-glc  (2-1) α-L-rha | H | H | OCH_3_ |
| **60** | OH | H | *O*-β-D-glc  (2-1) α-L-rha | H | OCH_3_ | H |
| **61** | OCH_3_ | O-CH_2_-O | | OCH_3_ | H | H |

| No. | **R_1_** | **R_2_** | **R_3_** | **R_4_** | **R_5_** | **R_6_** |
| --- | --- | --- | --- | --- | --- | --- |
| **82** | H | OH | H | OH | CH_3_ | CH_3_ |
| **83** | OH | OH | H | OH | CH_3_ | CH_3_ |
| **84** | H | OH | H | OH | CH_3_ | CH_2_OH |
| **85** | H | *O*-β-D-xyl | H | OH | CHO | CH_3_ |
| **86** | H | *O*-β-D-xyl | H | OH | CH_3_ | CH_2_OH |
| **87** | OH | C | OH | *O*-β-D-glc (6-1) β-D  -glc (4-1) α-L-rha | CH_3_ | CH_2_OH |
| **88** | H | *O*-α-L-ara | H | *O*-β-D-glc (6-1) β-D  -glc (4-1) α-L-rha | CH_3_ | CH_2_OH |
| **89** | H | *O*-α-L-ara | H | *O*-β-D-glc (6-1) β-D-glc | CH_3_ | CH_3_ |

**Fig. 1.** The structures of compounds **1**–**132** from *Elsholtzia*
