# Supplementary material for: Elsholtzia: phytochemistry and biological activities
Source: Chem Cent J. 2012 Dec 5;6:147. doi: 10.1186/1752-153X-6-147 (PMC3536681; doi:10.1186/1752-153X-6-147)
Supplement: Additional file 4 — Table S3. The volatile chemical components from Elsholtzia[41,43,44,47-49,51-79,106,114-118]. [file 1752-153X-6-147-S4.doc]

***Additional file 4: Table S3.*** *The volatile chemical components from Elsholtzia*

| **No.** | **Name** | **Formula** | **Source** | **Reference** |
| --- | --- | --- | --- | --- |
| 1 | linoleic acid ethyl ester | C_20_H_36_O_2_ | *E*. *bodinieri* | [41] |
| 2 | lauryl alcohol | C_12_H_26_O | *E*. *blanda* | [43] |
| 3 | monanic acid ethyl ester | C_11_H_22_O_2_ | *E*. *blanda* | [44] |
| 4 | octadecanoic acid ethyl ester | C_20_H_40_O_2_ | *E*. *blanda* | [44] |
| 5 | 1,4-cyclohexadiene | C_6_H_8_ | *E*. *densa* | [47] |
| 6 | 1,4-eicosadiene | C_22_H_42_ | *E*. *densa* | [47] |
| 7 | 1,8-cineolic acid | C_10_H_16_O | *E*. *densa* | [47] |
| 8 | 1-nonen-3-one | C_9_H_16_O | *E*. *densa* | [47] |
| 9 | 1-ter-2,4-diphenylpyrrole | C_20_H_21_N | *E*. *densa* | [47] |
| 10 | 6-methyl-2-phenylquindine | C_16_H_13_N | *E*. *densa* | [47] |
| 11 | 9,11-octadecadiene | C_20_H_42_ | *E*. *densa* | [47] |
| 12 | a-lonone | C_13_H_20_O | *E*. *densa* | [47] |
| 13 | elemicin | C_12_H_16_O_2_ | *E*. *densa* | [47] |
| 14 | elemol | C_15_H_26_O | *E*. *densa* | [47] |
| 15 | cis-△8-menthene | C_10_H_18_ | *E*. *densa* | [47] |
| 16 | citronellol | C_10_H_20_O | *E*. *densa* | [47] |
| 17 | myristic ether | C_11_H_12_O_3_ | *E*. *densa* | [47] |
| 18 | nordidiol | C_15_H_26_O | *E*. *densa* | [47] |
| 19 | 2,5-dimethyl dodecane | C_14_H_30_ | *E*. *densa* | [47] |
| 20 | 2-benzoyl-benzoic acid | C_14_H_10_O_3_ | *E*. *densa* | [47] |
| 21 | β-aromadendrene | C_15_H_24_ | *E*. *densa* | [47] |
| 22 | 6-eicosanone | C_20_H_40_O | *E*. *densa* | [47] |
|  |  |  | *E*. *stauntonii* | [47] |
| 23 | trans-geranylacetate | C_12_H_18_O_2_ | *E*. *stauntonii* | [47] |
| 24 | 2-methylene-6-methyl-5,7-octadien-3-ol | C_10_H_16_O | *E*. *stauntonii* | [47] |
| 25 | δ-guaiene | C_15_H_24_ | *E*. *stauntonii* | [47] |
| 26 | z-14-methyl-16-oxabicyclo [1,3,10] hexadec-13-ene | C_16_H_28_O | *E*. *stauntonii* | [47] |
| 27 | myrtenal | C_10_H_14_O | *E*. *stauntonii* | [47] |
| 28 | n-borhylene | C_10_H_16_ | *E*. *stauntonii* | [47] |
| 29 | neophadiene | C_20_H_28_ | *E*. *stauntonii* | [47] |
| 30 | pulegone | C_10_H_16_O | *E*. *stauntonii* | [47] |
| 31 | terpinyl acetate | C_12_H_20_O_2_ | *E*. *stauntonii* | [47] |
| 32 | cis-octahydro-1H-inden-1-one | C_9_H_14_O | *E*. *stauntonii* | [47] |
| 33 | eicosyl cyclohexane | C_26_H_52_ | *E*. *stauntonii* | [47] |
| 34 | elnemicin | C_12_H_16_O_3_ | *E*. *stauntonii* | [47] |
| 35 | hexadcanoic acid ethylester | C_18_H_36_O_2_ | *E*. *stauntonii* | [47] |
| 36 | isopinocamphone | C_10_H_16_O | *E*. *stauntonii* | [47] |
| 37 | 1,2-benzendicar boxylic acid dibutyl ester | C_16_H_22_O_4_ | *E. stauntonii* | [47] |
|  |  |  | *E*. *blanda* | [51] |
| 38 | 1a, 9a-dimethyl-cis-bicyclo [4,1,0] non-7-en-2-one | C_11_H_16_O | *E*. *stauntonii* | [47] |
|  |  |  | *E*. *blanda* | [51] |
| 39 | 2,4-hexadienoic acid-4′-methyl phenyl ester | C_13_H_14_O_2_ | *E*. *stauntonii i* | [47,71] |
|  |  |  | *E*. *blanda* | [51] |
| 40 | 2-methyl-5-acelylfuran | C_7_H_8_O_2_ | *E*. *densa* | [47,58] |
| 41 | 2-(2´, 3´-epoxy-3´-methyl butyl)-3-methyl furan | C_10_H_14_O_2_ | *E*. *stauntonii* | [47,71] |
| 42 | 3-(3,6-dimethyl-2-methoxyphenyl)-methylbutunal | C_14_H_20_O_2_ | *E*. *stauntonii* | [47,71] |
| 43 | β-ylangene | C_15_H_24_ | *E*. *stauntonii* | [47,71] |
| 44 | α-bergamotene | C_15_H_24_ | *E*. *stauntonii* | [47] |
|  |  |  | *E*. *blanda* | [51] |
|  |  |  | *E*. *frulicosa* | [57] |
|  |  |  | *E*. *splendens* | [73] |
| 45 | aromadondrene | C_15_H_26_ | *E*. *densa* | [47] |
|  |  |  | *E*. *calycoarpa* | [53] |
|  |  |  | *E*. *patrini* | [64] |
|  |  |  | *E*. *ciliata* | [65] |
|  |  |  | *E*. *enistacha* | [69] |
| 46 | αγ-curcumene | C_15_H_22_ | *E*. *stauntonii* | [47,71] |
|  |  |  | *E*. *blanda* | [51] |
| 47 | β-caryophyllene | C_15_H_24_ | *E*. *densa* | [47] |
|  |  |  | *E*. *bodinieri* | [48,72] |
|  |  |  | *E*. *splendens* | [55] |
|  |  |  | *E*. *stauntonii* | [60] |
|  |  |  | *E*. *blanda* | [63] |
|  |  |  | *E*. *ciliata* | [65- 67] |
|  |  |  | *E*. *enistacha* | [69] |
|  |  |  | *E*. *polystachya* | [70] |
| 48 | benzyl benzoate | C_14_H_12_O_2_ | *E*. *stauntonii* | [47,71] |
|  |  |  | *E*. *blanda* | [51] |
| 49 | β-eudesmol | C_15_H_26_O | *E*. *densa* | [47] |
|  |  |  | *E*. *stauntonii* | [47] |
|  |  |  | *E*. *blanda* | [51] |
|  |  |  | *E*. *splendens* | [55] |
| 50 | farnesol | C_15_H_26_O | *E*. *densa* | [47] |
| 51 | santalene | C_15_H_24_ | *E*. *densa* | [47] |
| 52 | β-lonone | C_13_H_20_O | *E*. *densa* | [47] |
|  |  |  | *E*. *bodinieri* | [72] |
| 53 | β-terpilneol | C_10_H_18_O | *E*. *densa* | [47,58] |
|  |  |  | *E*. *blanda* | [63] |
| 54 | camphorquinone | C_10_H_14_O_2_ | *E*. *stauntonii* | [47,59,71] |
|  |  |  | *E*. *densa* | [58] |
| 55 | cedrol | C_15_H_26_O | *E*. *densa* | [47,58] |
|  |  |  | *E*. *splendens* | [55] |
| 56 | β-sitosterol | C_29_H_50_O | *E*. *bodinieri* | [48] |
| 57 | 2, 5-dimethyl-3-methylene-1, 5-heptadiene | C_10_H_16_ | *E*. *bodinieri* | [48] |
| 58 | eriophillene | C_15_H_24_ | *E*. *bodinieri* | [48] |
| 59 | dibutyl phthalate | C_16_H_22_O_4_ | *E*. *bodinieri* | [48] |
| 60 | epi-curzerenone | C_15_H_18_O_2_ | *E*. *bodinieri* | [48] |
| 61 | isoledene | C_15_H_24_ | *E*. *bodinieri* | [48] |
| 62 | ligustilide | C_12_H_14_O_2_ | *E*. *bodinieri* | [48] |
| 63 | ethyl laurate | C_24_H_48_O_2_ | *E*. *bodinieri* | [48] |
| 64 | (+)-4-carene | C_10_H_16_ | *E*. *bodinieri* | [48] |
| 65 | elsholtzione | C_10_H_14_O_2_ | *E*. *bodinieri* | [48] |
| 66 | isothymol | C_10_H_14_O | *E*. *bodinieri* | [48] |
|  |  |  | *E*. *splendens* | [55] |
| 67 | β-caryophyllene oxide | C_15_H_24_O | *E*. *bodinieri* | [48] |
|  |  |  | *E*. *stauntonii* | [60] |
| 68 | 2-butenylbenzene | C_10_H_12_ | *E*. *bodinieri* | [48,72] |
| 69 | α-myrcene | C_10_H_16_ | *E*. *bodinieri* | [48,72] |
| 70 | β-citronellol | C_10_H_20_O | *E*. *bodinieri* | [48,72] |
| 71 | β-copaene | C_15_H_24_ | *E*. *bodinieri* | [48,72] |
| 72 | behenic acid, ethyl ester | C_24_H_48_O_2_ | *E*. *bodinieri* | [48,72] |
| 73 | (+) spathulenol | C_15_H_24_O | *E*. *bodinieri* | [48,72] |
| 74 | 1, 1-diethoxyethane | C_6_H_14_O_2_ | *E*. *bodinieri* | [48,72] |
| 75 | cineole | C_10_H_18_O | *E*. *bodinieri* | [48,49,72,76] |
| 76 | elsholtzitol | C_10_H_16_O_2_ | *E*. *bodinieri* | [48,72] |
|  |  |  | *E. calycoarpa* | [53] |
| 77 | heptacosane | C_27_H_56_ | *E*. *bodinieri* | [48,72] |
| 78 | eugenol | C_10_H_12_O_2_ | *E*. *bodinieri* | [48,75] |
| 79 | germacrone | C_15_H_22_O | *E*. *bodinieri* | [48,72] |
| 80 | isocaryophyllene | C_15_H_24_ | *E*. *bodinieri* | [48,72] |
| 81 | linoleic acid | C_18_H_32_O_2_ | *E*. *bodinieri* | [48,72] |
| 82 | δ-cadinene | C_15_H_24_ | *E*. *bodinieri* | [48,72] |
| 83 | z-β-terpineol | C_10_H_18_O | *E*. *bodinieri* | [48,72] |
| 84 | palmitic acid | C_16_H_32_O_2_ | *E*. *enistacha* | [48] |
|  |  |  | *E*. *bodinieri* | [75] |
| 85 | α-pinene | C_10_H_16_ | *E*. *bodinieri* | [48,49] |
|  |  |  | *E*. *blanda* | [50,51,62,63] |
|  |  |  | *E*. *penduliflora* | [52] |
|  |  |  | *E*. *calycoarpa* | [53] |
|  |  |  | *E*. *feddei* | [54] |
|  |  |  | *E*. *splendens* | [55,56] |
|  |  |  | *E*. *frulicosa* | [57] |
|  |  |  | *E*. *densa* | [58] |
|  |  |  | *E*. *stauntonii* | [59,60,71] |
|  |  |  | *E*. *strobilifera* | [61] |
|  |  |  | *E*. *patrini* | [64] |
|  |  |  | *E*. *ciliata* | [65-67] |
|  |  |  | *E*. *rugulosa* | [68] |
|  |  |  | *E*. *enistacha* | [69] |
|  |  |  | *E*. *polystachya* | [70] |
| 86 | β-myrcene | C_10_H_16_ | *E*. *bodinieri* | [48] |
|  |  |  | *E*. *feddei* | [54] |
|  |  |  | *E*. *frulicosa* | [57] |
|  |  |  | *E*. *stauntonii* | [60,71] |
|  |  |  | *E*. *ciliata* | [65,67] |
| 87 | β-pinene | C_10_H_16_ | *E*. *bodinieri* | [48,49,72] |
|  |  |  | *E*. *blanda* | [50,51,62,63] |
|  |  |  | *E*. *penduliflora* | [52] |
|  |  |  | *E*. *splendens* | [55,56,73] |
|  |  |  | *E*. *frulicosa* | [57] |
|  |  |  | *E*. *stauntonii* | [59,60,71] |
|  |  |  | *E*. *strobilifera* | [61] |
|  |  |  | *E*. *patrini* | [64] |
|  |  |  | *E*. *ciliata* | [65,67] |
|  |  |  | *E*. *rugulosa* | [68] |
|  |  |  | *E*. *enistacha* | [69] |
|  |  |  | *E*. *polystachya* | [70] |
| 88 | β-terpinene | C_10_H_16_ | *E*. *bodinieri* | [48,72] |
|  |  |  | *E. stauntonii* | [60] |
|  |  |  | *E*. *patrini* | [64] |
| 89 | β-ionone | C_13_H_20_O | *E*. *bodinieri* | [48] |
|  |  |  | *E*. *splendens* | [55] |
| 90 | borneol | C_10_H_18_O | *E*. *bodinieri* | [48,70,72] |
|  |  |  | *E*. *blanda* | [50,62,63] |
|  |  |  | *E*. *splendens* | [55] |
| 91 | linalool | C_10_H_18_O | *E*. *bodinieri* | [48] |
|  |  |  | *E*. *penduliflora* | [52] |
| 92 | thymol | C_10_H_14_O | *E*. *bodinieri* | [48] |
|  |  |  | *E*. *penduliflora* | [52] |
| 93 | trans-pinocarveol | C_10_H_16_O | *E*. *bodinieri* | [49] |
| 94 | 3-thujen-2-ol | C_10_H_16_O | *E*. *bodinieri* | [49] |
| 95 | 1,3,4-trimethyl-2-metoxybenzene | C_10_H_14_O_2_ | *E*. *bodinieri* | [49] |
| 96 | terpinene | C_10_H_16_ | *E*. *bodinieri* | [49] |
| 97 | campholenic aldehyde | C_10_H_16_O | *E*. *bodinieri* | [49] |
| 98 | (+)-norinone | C_9_H_14_O | *E*. *bodinieri* | [49] |
| 99 | σ-cymene | C_10_H_14_ | *E*. *bodinieri* | [49] |
| 100 | pinocarvone | C_10_H_14_O | *E*. *bodinieri* | [49] |
|  |  |  | *E*. *strobilifera* | [61] |
| 101 | camphene | C_10_H_16_ | *E*. *bodinieri* | [49,75] |
|  |  |  | *E*. *blanda* | [50,63] |
|  |  |  | *E*. *penduliflora* | [52] |
|  |  |  | *E*. *feddei* | [54] |
|  |  |  | *E*. *splendens* | [55,56,73] |
|  |  |  | *E*. *frulicosa* | [57] |
|  |  |  | *E*. *stauntonii* | [59,71] |
|  |  |  | *E*. *strobilifera* | [61] |
|  |  |  | *E*. *patrini* | [64] |
|  |  |  | *E*. *ciliata* | [65] |
| 102 | sabinene hydrate | C_10_H_18_O | *E*. *blanda* | [50] |
| 103 | terpineol | C_10_H_18_O | *E*. *blanda* | [50] |
| 104 | α-terpinolene | C_10_H_16_ | *E*. *blanda* | [50] |
| 105 | bergapten | C_12_H_8_O_4_ | *E*. *blanda* | [50] |
|  |  |  | *E*. *calycoarpa* | [53] |
| 106 | α-terpinene | C_10_H_16_ | *E*. *blanda* | [50,63] |
|  |  |  | *E*. *stauntonii* | [60] |
|  |  |  | *E*. *strobilifera* | [61] |
|  |  |  | *E*. *ciliata* | [65] |
|  |  |  | *E*. *enistacha* | [69] |
|  |  |  | *E*. *frulicosa* | [112] |
| 107 | γ-terpinene | C_10_H_16_ | *E*. *bodinieri* | [49] |
|  |  |  | *E*. *penduliflora* | [52] |
| 108 | β-ocimene | C_15_H_24_ | *E*. *blanda* | [50,63] |
|  |  |  | *E*. *ciliata* | [67] |
| 109 | bornyl acetate | C_12_H_20_O_2_ | *E*. *blanda* | [50,62] |
|  |  |  | *E*. *splendens* | [55] |
|  |  |  | *E*. *stauntonii* | [71] |
| 110 | 2,3-dihydrofuranone | C_4_H_6_O_2_ | *E*. *blanda* | [51] |
| 111 | 2-methyl-5-(1-methyl ethyl)-phenol | C_10_H_14_O | *E*. *blanda* | [51] |
| 112 | 3-nonen-2-one | C_9_H_16_O | *E*. *blanda* | [51] |
| 113 | 5-methl-3-hexen-2-one | C_7_H_12_O | *E*. *blanda* | [51] |
| 114 | cannabivarin | C_19_H_22_O_2_ | *E*. *blanda* | [51] |
| 115 | β-tocopherol | C_28_H_48_O_2_ | *E*. *blanda* | [51] |
| 116 | bulnesol | C_15_H_26_O | *E*. *blanda* | [51] |
| 117 | copaene | C_15_H_24_ | *E*. *blanda* | [51] |
| 118 | cyclododecanone | C_12_H_22_O | *E*. *blanda* | [51] |
| 119 | E-11,13-dimethyl-12-tetradecen-1-ol-acetate | C_18_H_34_O_2_ | *E*. *blanda* | [51] |
| 120 | farnesyl acetate | C_17_H_28_O_2_ | *E*. *blanda* | [51] |
| 121 | furanmethaol | C_5_H_6_O_2_ | *E*. *blanda* | [51] |
| 122 | santene | C_9_H_14_ | *E*. *blanda* | [51] |
| 123 | tetradecane | C_14_H_30_ | *E*. *blanda* | [51] |
| 124 | 4-phenylbutene-1 | C_10_H_12_ | *E*. *penduliflora* | [52] |
|  |  |  | *E*. *splendens* | [73] |
| 125 | limonene | C_10_H_16_ | *E*. *penduliflora* | [52] |
| 126 | myrcene | C_10_H_16_ | *E*. *penduliflora* | [52] |
| 127 | naphthalene | C_10_H_8_ | *E*. *penduliflora* | [52] |
| 128 | sabinene | C_10_H_16_ | *E*. *penduliflora* | [52] |
| 129 | Γ-elemene | C_15_H_24_ | *E*. *penduliflora* | [52] |
| 130 | trans-caryophyllene | C_15_H_24_ | *E*. *penduliflora* | [52] |
| 131 | humulene | C_15_H_24_ | *E*. *penduliflora* | [52] |
| 132 | 4-phenylbutene-1 | C_10_H_12_ | *E*. *penduliflora* | [52] |
|  |  |  | *E*. *splendens* | [73] |
| 133 | β-cubebene | C_15_H_24_ | *E*. *penduliflora* | [52] |
|  |  |  | *E*. *strobilifera* | [61] |
|  |  |  | *E*. *ciliata* | [65,67] |
|  |  |  | *E*. *stauntonii* | [71] |
|  |  |  | *E*. *splendens* | [73] |
| 134 | β-dehydro elsholizione | C_10_H_12_O_2_ | *E*. *penduliflora* | [52] |
|  |  |  | *E*. *patrini* | [64] |
|  |  |  | *E*. *ciliata* | [66,79] |
| 135 | 2-(2′,3′-dimethylbutyl)-3-methyl furan | C_11_H_18_O | *E*. *calycoarpa* | [53] |
| 136 | 2-butanone | C_4_H_8_O | *E*. *calycoarpa* | [53] |
| 137 | anethole | C_10_H_12_O | *E*. *calycoarpa* | [53] |
| 138 | furfural | C_5_H_4_O_2_ | *E*. *calycoarpa* | [53] |
| 139 | longifolene | C_15_H_24_ | *E*. *calycoarpa* | [53] |
| 140 | methyl isopropyl ketone | C_5_H_10_O | *E*. *calycoarpa* | [53] |
| 141 | myrtenol | C_10_H_16_O | *E*. *calycoarpa* | [53] |
| 142 | patchoulene | C_15_H_24_ | *E*. *calycoarpa* | [53] |
| 143 | (+)-epi-bicyclosesquiphellandrtene | C_10_H_14_ | *E*. *feddei* | [54] |
| 144 | 1-(1-oxobutyl)-1,2-dihydropyridin | C_9_H_16_ON | *E*. *feddei* | [54] |
| 145 | 1,2,3,4,4a,5,6,8a-octahydro-naphthalene | C_10_H_16_ | *E*. *feddei* | [54] |
| 146 | 1,2,3,5,6,7,8,8a-octahydro-naphthalene | C_10_H_16_ | *E*. *feddei* | [54] |
| 147 | 1,3,6-octatriene, 3,7-dimethyl- | C_10_H_14_ | *E*. *feddei* | [54] |
| 148 | 1,6-octadien-3-ol, 3,7-dimethyl- | C_11_H_18_O | *E*. *feddei* | [54] |
| 149 | 1-adamantanol | C_10_H_14_O | *E*. *feddei* | [54] |
| 150 | 1-benzene, 1-methyl-4-(1-methylethyl) | C_9_H_14_ | *E*. *feddei* | [54] |
| 151 | 2-fluorophenyl isocyanate | C_7_H_6_NF | *E*. *feddei* | [54] |
| 152 | 2-hydroxy-phenylethyl-benzoic acid | C_14_H_12_O_3_ | *E*. *feddei* | [54] |
| 153 | 2-hydroxy-5-methylbenzaldehyde | C_8_H_8_O_2_ | *E*. *feddei* | [54] |
| 154 | 2-cyclopenten-1-one, 3-methyl- | C_7_H_10_O | *E*. *feddei* | [54] |
| 155 | 2,6-octadien-1-ol, 3,7-dimethyl- | C_11_H_18_O | *E*. *feddei* | [54] |
| 156 | 2,7-dimethyl-1,3,7-octatriene | C_8_H_14_ | *E*. *feddei* | [54] |
| 157 | 2,4-hexadiene | C_6_H_8_ | *E*. *feddei* | [54] |
| 158 | 2-methyl-5-(1-methyl)- cyclohexen-1-one | C_10_H_16_O | *E*. *feddei* | [54] |
| 159 | 5-ethylidene-1-methyl-cycloheptene | C_10_H_16_ | *E*. *feddei* | [54] |
| 160 | 5,9,13-pentadecatrien-2-one | C_15_H_18_O | *E*. *feddei* | [54] |
| 161 | 6,10,14-trimethyl-pentadecanone | C_15_H_36_O | *E*. *feddei* | [54] |
| 162 | 7-oxabicyclo [4.1.0]heptane, 1-methyl- | C_7_H_12_O | *E*. *feddei* | [54] |
| 163 | 7,11-dimethyl-1,6,10-dodecatrinen | C_14_H_24_ | *E*. *feddei* | [54] |
| 164 | bicyclo[3.1.0]hex-2-ene, 2-methyl- | C_6_H_10_ | *E*. *feddei* | [54] |
| 165 | bicyclo[3.1.0]hex-2-ene, 4-methyl- | C_6_H_10_ | *E*. *feddei* | [54] |
| 166 | bicyclo[3.1.1]hept-2-ene, 2,6-dimethyl- | C_11_H_18_O | *E*. *feddei* | [54] |
| 167 | bicyclo[3.1.1]heptane,6,6-dimethyl- | C_9_H_16_ | *E*. *feddei* | [54] |
| 168 | bicyclo[4.4.0] dec-1-ene, 2-isoproply- | C_13_H_22_ | *E*. *feddei* | [54] |
| 169 | β-sesquiphellandrtene | C_10_H_16_ | *E*. *feddei* | [54] |
| 170 | butyl-1,2-benzenedicarboxylic acid | C_12_H_14_O | *E*. *feddei* | [54] |
| 171 | D-germacrene | C_10_H_16_ | *E*. *feddei* | [54] |
| 172 | D-limonene | C_10_H_16_ | *E*. *feddei* | [54] |
| 173 | docasane | C_22_H_46_ | *E*. *feddei* | [54] |
| 174 | heptadecane | C_17_H_36_ | *E*. *feddei* | [54] |
| 175 | hexatriacontane | C_36_H_74_ | *E*. *feddei* | [54] |
| 176 | isopulegol | C_10_H_20_O | *E*. *feddei* | [54] |
| 177 | octacosane | C_28_H_58_ | *E*. *feddei* | [54] |
| 178 | O-menth-8-ene | C_10_H_18_ | *E*. *feddei* | [54] |
| 179 | phenol, 2,3,5,6-tertramethyl- | C_10_H_14_O | *E*. *feddei* | [54] |
| 180 | trans-limonene oxide | C_10_H_16_O | *E*. *feddei* | [54] |
| 181 | tricosane | C_23_H_48_ | *E*. *feddei* | [54] |
| 182 | 4-carene | C_10_H_16_ | *E*. *feddei* | [54] |
| 183 | 2,4-dimethyl anisole | C_9_H_12_O | *E*. *feddei* | [54] |
|  |  |  | *E*. *ciliata* | [66] |
| 184 | 6-methyl-hept-5-en-2-one | C_8_H_14_O | *E*. *splendens* | [55] |
| 185 | 2-methyl butyrate | C_6_H_12_O_2_ | *E*. *splendens* | [55] |
| 186 | bisabolol | C_15_H_26_O | *E*. *splendens* | [55] |
| 187 | β-sinensal | C_15_H_22_O | *E*. *splendens* | [55] |
| 188 | β-thujone | C_10_H_16_O | *E*. *splendens* | [55] |
| 189 | carvone | C_10_H_14_O | *E*. *splendens* | [55] |
| 190 | cedrenol | C_10_H_24_O | *E*. *splendens* | [55] |
| 191 | carvone oxide | C_10_H_14_O_2_ | *E*. *splendens* | [55] |
| 192 | β-elemol | C_15_H_26_O | *E*. *splendens* | [55] |
| 193 | trans-carveol | C_10_H_16_O | *E*. *splendens* | [55] |
| 194 | trans-caryophyllene epoxide | C_15_H_24_O | *E*. *splendens* | [55] |
| 195 | trans,trans-fanesol | C_15_H_26_O | *E*. *splendens* | [55] |
| 196 | tridecane | C_13_H_28_ | *E*. *splendens* | [55] |
| 197 | undecanal | C_11_H_22_O | *E*. *splendens* | [55] |
| 198 | undecane | C_11_H_24_ | *E*. *splendens* | [55] |
| 199 | undecanoic acid | C_11_H_22_O_2_ | *E*. *splendens* | [55] |
| 200 | viridiflorol | C_15_H_26_O | *E*. *splendens* | [55] |
| 201 | δ-3-carene | C_10_H_16_ | *E*. *splendens* | [55] |
| 202 | γ-eudesmol | C_15_H_26_O | *E*. *splendens* | [55] |
| 203 | cedry acetate | C_17_H_28_O_2_ | *E*. *splendens* | [55] |
| 204 | cis-3-hexenal | C_6_H_10_O | *E*. *splendens* | [55] |
| 205 | cis-caryophyllene epoxide | C_15_H_24_O | *E*. *splendens* | [55] |
| 206 | cis, trans-farnesol | C_15_H_26_O | *E*. *splendens* | [55] |
| 207 | cis-linalool oxide | C_10_H_18_O_2_ | *E*. *splendens* | [55] |
| 208 | citronellal | C_10_H_18_O | *E*. *splendens* | [55] |
| 209 | citronellyl acetate | C_12_H_22_O_2_ | *E*. *splendens* | [55] |
| 210 | citronellyl formate | C_10_H_20_O_2_ | *E*. *splendens* | [55] |
| 211 | dehydro carveol | C_10_H_14_O | *E*. *splendens* | [55] |
| 212 | dodecanoic acid | C_12_H_24_O_2_ | *E*. *splendens* | [55] |
| 213 | ethyl heptadecanoate | C_19_H_38_O_2_ | *E*. *splendens* | [55] |
| 214 | eugenol methyl ether | C_11_H_14_O_2_ | *E*. *splendens* | [55] |
| 215 | geranial | C_10_H_16_O | *E*. *splendens* | [55] |
| 216 | geraniol | C_10_H_18_O | *E*. *splendens* | [55] |
| 217 | germacrene-d | C_15_H_24_ | *E*. *splendens* | [55] |
| 218 | geranyl acetate | C_13_H_20_O_2_ | *E*. *splendens* | [55] |
| 219 | globulol | C_15_H_26_O | *E*. *splendens* | [55] |
| 220 | heptyl acetate | C_9_H_18_O_2_ | *E*. *splendens* | [55] |
| 221 | hexadecanal | C_16_H_32_O | *E*. *splendens* | [55] |
| 222 | isopiperitone | C_10_H_16_O | *E*. *splendens* | [55] |
| 223 | lauric acid | C_12_H_24_O_2_ | *E*. *splendens* | [55] |
| 224 | menthol | C_10_H_20_O | *E*. *splendens* | [55] |
| 225 | methyl octadecanoate | C_19_H_38_O_2_ | *E*. *splendens* | [55] |
| 226 | murrolene | C_15_H_24_ | *E*. *splendens* | [55] |
| 227 | myrcene epoxide | C_10_H_16_O | *E*. *splendens* | [55] |
| 228 | myrcenol | C_10_H_18_O | *E*. *splendens* | [55] |
| 229 | nerol | C_10_H_18_O | *E*. *splendens* | [55] |
| 230 | neryl acetate | C_12_H_20_O_2_ | *E*. *splendens* | [55] |
| 231 | n-hexanal | C_6_H_12_O | *E*. *splendens* | [55] |
| 232 | octadecanoic acid | C_18_H_36_O_2_ | *E*. *splendens* | [55] |
| 233 | octadecanol | C_18_H_38_O | *E*. *splendens* | [55] |
| 234 | octanal | C_8_H_16_O | *E*. *splendens* | [55] |
| 235 | octanoic acid | C_8_H_16_O_2_ | *E*. *splendens* | [55] |
| 236 | perilla alcohol | C_10_H_16_O | *E*. *splendens* | [55] |
| 237 | perilla acetate | C_12_H_18_O_2_ | *E*. *splendens* | [55] |
| 238 | p-mentha-1,8-dien-10-ol | C_10_H_16_O | *E*. *splendens* | [55] |
| 239 | sesquiphellandrene | C_15_H_24_ | *E*. *splendens* | [55] |
| 240 | spathulenol | C_15_H_24_O | *E*. *splendens* | [55] |
| 241 | terpinolene | C_10_H_16_ | *E*. *splendens* | [55] |
| 242 | tetradecanal | C_14_H_28_O | *E*. *splendens* | [55] |
| 243 | trans-2-hexenal | C_6_H_10_O | *E*. *splendens* | [55] |
| 244 | 2,4-hexadienal | C_6_H_8_O | *E*. *splendens* | [55] |
| 245 | cymene | C_10_H_14_ | *E*. *splendens* | [56] |
| 246 | α-trans-bergapten | C_12_H_8_O_4_ | *E*. *splendens* | [56] |
| 247 | isobornyl acetate | C_12_H_20_O_2_ | *E*. *blanda* | [56] |
| 248 | 2-methyl-4-actyl-cyclohexano | C_10_H_16_O | *E*. *frulicosa* | [57] |
| 249 | 5-neo-cedranol | C_15_H_26_O | *E*. *frulicosa* | [57] |
| 250 | 2-octene-1-ol | C_8_H_14_O | *E*. *frulicosa* | [57] |
| 251 | 9-octadecenoic acid | C_18_H_34_O_2_ | *E*. *frulicosa* | [57] |
| 252 | 9-octadecenoic acid methyl ester | C_19_H_36_O_2_ | *E*. *frulicosa* | [57] |
| 253 | acetic acid dodecyl ester | C_14_H_28_O_2_ | *E*. *frulicosa* | [57] |
| 254 | cis-caryophyllene | C_15_H_24_ | *E*. *frulicosa* | [57] |
| 255 | diethyl phthalate | C_12_H_14_O_4_ | *E*. *frulicosa* | [57] |
| 256 | dodecanene | C_12_H_24_O | *E*. *frulicosa* | [57] |
| 257 | hexcadecane | C_16_H_34_ | *E*. *frulicosa* | [57] |
| 258 | octenyl acetate | C_10_H_18_O_2_ | *E*. *frulicosa* | [57] |
| 259 | pentadecanol acetate | C_17_H_34_O_2_ | *E*. *frulicosa* | [57] |
| 260 | perillene | C_10_H_14_O | *E*. *frulicosa* | [57] |
| 261 | thujene | C_10_H_16_ | *E*. *frulicosa* | [57] |
|  |  |  | *E*. *strobilifera* | [61] |
| 262 | 1-ethyl-3,5-dimethylbenzene | C_10_H_14_ | *E*. *densa* | [58] |
| 263 | 2-methyl borneol | C_11_H_20_O | *E*. *densa* | [58] |
| 264 | 3-methyl-2-cyclopentene-1-one | C_6_H_8_O | *E*. *densa* | [58] |
| 265 | 9,12-octadecadienoc acid | C_18_H_32_O_2_ | *E*. *densa* | [58] |
| 266 | eicosane | C_20_H_42_ | *E*. *densa* | [58] |
| 267 | cumic acid | C_10_H_12_O_2_ | *E*. *densa* | [58] |
| 268 | hydroxyl citronellol | C_10_H_22_O_2_ | *E*. *densa* | [58] |
| 269 | lapachol | C_15_H_14_O_3_ | *E*. *densa* | [58] |
| 270 | phenethyl alcohol | C_8_H_10_O | *E*. *densa* | [58] |
| 271 | p-metha-1,5-dien-8-ol | C_10_H_16_O | *E*. *densa* | [58] |
| 272 | trans-△8-menthene | C_10_H_18_ | *E*. *densa* | [58] |
| 273 | benzene methanol | C_7_H_8_O | *E*. *densa* | [58] |
|  |  |  | *E*. *stauntonii* | [71] |
| 274 | 2-methyl-5-iso-pentanone-furane | C_10_H_14_O_2_ | *E*. *stauntonii* | [60] |
| 275 | 2-methyl-5-pentanone(4)-furane | C_10_H_14_O_2_ | *E*. *stauntonii* | [60] |
| 276 | 2-methyl-5-pentanone(3)-furane | C_10_H_14_O_2_ | *E*. *stauntonii* | [60] |
| 277 | α-caryophyllene oxide | C_15_H_24_O | *E*. *stauntonii* | [60] |
| 278 | 12-hydroxyl-3-carbonyl-8,11,13-dehydroabietane | C_20_H_26_O_2_ | *E*. *stauntonii* | [60] |
| 279 | 2-ethyl-5-iso-butanone-furane | C_10_H_14_O_2_ | *E*. *stauntonii* | [60] |
| 280 | amyrin | C_15_H_26_O | *E*. *stauntonii* | [60] |
| 281 | cadindiene | C_15_H_24_ | *E*. *stauntonii* | [60] |
| 282 | trans-linalool oxide | C_10_H_18_O_2_ | *E*. *stauntonii* | [60] |
| 283 | norprist- 2- one | C_18_H_36_O | *E*. *stauntonii* | [60] |
| 284 | ocimene | C_15_H_24_ | *E*. *stauntonii* | [60] |
| 285 | trans-sabinene | C_10_H_16_ | *E*. *stauntonii* | [60] |
| 286 | 4-isopropylbenzyl alcohol | C_10_H_14_O | *E*. *stauntonii* | [60] |
| 287 | 1-epi-cubenol | C_15_H_26_O | *E*. *strobilifera* | [61] |
| 288 | 3-octanol acetate | C_10_H_20_O_2_ | *E*. *strobilifera* | [61] |
| 289 | 3-thujyl acetate | C_12_H_20_O_2_ | *E*. *strobilifera* | [61] |
| 290 | 9-epi-β-caryophyllene | C_15_H_24_ | *E*. *strobilifera* | [61] |
| 291 | a-acoradiene | C_15_H_24_ | *E*. *strobilifera* | [61] |
| 292 | ar-curcumene | C_15_H_22_ | *E*. *strobilifera* | [61] |
| 293 | apiol | C_12_H_14_O_4_ | *E*. *strobilifera* | [61] |
| 294 | aromadendrene isomer | C_15_H_24_ | *E*. *strobilifera* | [61] |
| 295 | α-Z-damascone | C_13_H_20_O | *E*. *strobilifera* | [61] |
| 296 | β-cedrene | C_15_H_24_ | *E*. *strobilifera* | [61] |
| 297 | β-gurjunene | C_15_H_24_ | *E*. *strobilifera* | [61] |
| 298 | *trans*-myrtenol acetate | C_12_H_18_O_2_ | *E*. *strobilifera* | [61] |
| 299 | trans-sabinene hydrate | C_10_H_18_O | *E*. *strobilifera* | [61] |
| 300 | tricyclene | C_10_H_16_ | *E*. *strobilifera* | [61] |
| 301 | valerianol | C_15_H_26_O | *E*. *strobilifera* | [61] |
| 302 | verbenone | C_10_H_14_O | *E*. *strobilifera* | [61] |
| 303 | γ-bisabolene | C_15_H_24_ | *E*. *strobilifera* | [61] |
| 304 | (Z)-ocimene | C_15_H_24_ | *E*. *strobilifera* | [61] |
| 305 | pinocamphone | C_10_H_16_O | *E*. *strobilifera* | [61] |
| 306 | sabinol | C_10_H_16_O | *E*. *strobilifera* | [61] |
| 307 | thujone | C_10_H_16_O | *E*. *strobilifera* | [61] |
| 308 | trans-calamenene | C_15_H_22_ | *E*. *strobilifera* | [61] |
| 309 | trans-calamenen-10-ol | C_15_H_22_O | *E*. *strobilifera* | [61] |
| 310 | trans-carvyl acetate | C_12_H_18_O_2_ | *E*. *strobilifera* | [61] |
| 311 | sabinol | C_10_H_16_O | *E*. *strobilifera* | [61] |
| 312 | thujone | C_10_H_16_O | *E*. *strobilifera* | [61] |
| 313 | trans-calamenene | C_15_H_22_ | *E*. *strobilifera* | [61] |
| 314 | trans-calamenen-10-ol | C_15_H_22_O | *E*. *strobilifera* | [61] |
| 315 | trans-carvyl acetate | C_12_H_18_O_2_ | *E*. *strobilifera* | [61] |
| 316 | linalyl acetate | C_12_H_20_O_2_ | *E*. *strobilifera* | [61] |
| 317 | methyl geranate | C_11_H_18_O_2_ | *E*. *strobilifera* | [61] |
| 318 | muurolol | C_15_H_26_O | *E*. *strobilifera* | [61] |
| 319 | neoiso-dihydrocarveol | C_10_H_18_O | *E*. *strobilifera* | [61] |
| 320 | neral | C_10_H_16_O | *E*. *strobilifera* | [61] |
| 321 | n-tetradecane | C_14_H_30_ | *E*. *strobilifera* | [61] |
| 322 | cis-14-nor-muurol-5-en-4-one | C_14_H_22_O | *E*. *strobilifera* | [61] |
| 323 | cis-β-guaiene | C_15_H_24_ | *E*. *strobilifera* | [61] |
| 324 | cis-calamenen-10-ol | C_15_H_22_O | *E*. *strobilifera* | [61] |
| 325 | cis-carvyl acetate | C_12_H_18_O_2_ | *E*. *strobilifera* | [61] |
| 326 | cis-muurola-4(14),5-diene | C_15_H_24_ | *E*. *strobilifera* | [61] |
| 327 | dimethyl phthalate | C_10_H_10_O | *E*. *strobilifera* | [61] |
| 328 | (E)-ocimene | C_15_H_24_ | *E*. *strobilifera* | [61] |
| 329 | ethyl nerolate | C_10_H_20_O_2_ | *E*. *strobilifera* | [61] |
| 330 | fenchene | C_10_H_16_ | *E*. *strobilifera* | [61] |
| 331 | heilfolen-12-al A | C_15_H_22_O | *E*. *strobilifera* | [61] |
| 332 | humulene epoxide Ⅱ | C_15_H_24_O | *E*. *strobilifera* | [61] |
| 333 | iso-dihydrocarveol | C_10_H_18_O | *E*. *strobilifera* | [61] |
| 334 | isothujone | C_10_H_16_O | *E*. *strobilifera* | [61] |
| 335 | bourbonene | C_15_H_24_ | *E*. *strobilifera* | [61] |
|  |  |  | *E*. *blanda* | [63] |
| 336 | β-phellandrene | C_10_H_16_ | *E*. *strobilifera* | [61] |
|  |  |  | *E*. *ciliata* | [65] |
| 337 | α-cadinene | C_15_H_24_ | *E*. *strobilifera* | [61] |
|  |  |  | *E*. *ciliata* | [66] |
| 338 | α-copaene | C_15_H_24_ | *E*. *strobilifera* | [61] |
|  |  |  | *E*. *enistacha* | [69] |
| 339 | bornyl formate | C_11_H_18_O_2_ | *E*. *blanda* | [63] |
| 340 | piperitone | C_10_H_16_O | *E*. *blanda* | [63] |
| 341 | tricyclane | C_9_H_7_N_3_S | *E*. *blanda* | [63] |
| 342 | γ-muurolene | C_15_H_24_ | *E*. *blanda* | [63] |
| 343 | 4-methyl-2,6-diterbutyl-phenol | C_15_H_24_O | *E*. *patrini* | [64] |
| 344 | β-patchoulene | C_15_H_24_ | *E*. *patrini* | [64] |
| 345 | calarene | C_15_H_24_ | *E*. *patrini* | [64] |
| 346 | 2-methoxy-1,3,5-trimethylbenzene | C_10_H_14_O | *E*. *patrini* | [64] |
| 347 | p-(1,1-dimethyl propyl)phenol | C_11_H_16_O | *E*. *patrini* | [64] |
| 348 | △^3^-carene | C_10_H_16_ | *E*. *patrini* | [64] |
| 349 | mayurone | C_14_H_20_O | *E*. *patrini* | [64] |
| 350 | α, α,4-trimethyl benzenemethanol | C_10_H_14_O | *E*. *ciliata* | [65] |
| 351 | 2,3,3-trimethyl-1,4-pentadiene | C_10_H_14_ | *E*. *ciliata* | [65] |
| 352 | 2-ethylidene cycloheptene | C_9_H_14_ | *E*. *ciliata* | [65] |
| 353 | 2-methoxy-5-(1-propenyl)-phenoll | C_10_H_12_O_2_ | *E*. *ciliata* | [65] |
| 354 | 5-isopropyl-6-methyl-hepta-3,5-dien-2-ol | C_11_H_20_O | *E*. *ciliata* | [65] |
| 355 | 4-phenyl-2-butanone | C_10_H_12_O | *E*. *ciliata* | [65] |
| 356 | cadinene | C_15_H_24_ | *E*. *ciliata* | [65] |
| 357 | hexanoic acid | C_6_H_12_O_2_ | *E*. *ciliata* | [65] |
| 358 | longipinene | C_15_H_24_ | *E*. *ciliata* | [65] |
| 359 | methyl carvacryiether | C_11_H_16_O | *E*. *ciliata* | [65] |
| 360 | methyl jasmonate | C_13_H_20_O_3_ | *E*. *ciliata* | [65] |
| 361 | o-buthyl phthalate | C_16_H_22_O_4_ | *E*. *ciliata* | [65] |
| 362 | α-murrolene | C_15_H_24_ | *E*. *ciliata* | [65] |
|  |  |  | *E*. *polystachya* | [70] |
| 363 | 4-methyl-[1-methylethyl]-3-cyclohexen-1-ol | C_10_H_18_O | *E*. *ciliata* | [65] |
|  |  |  | *E. stauntonii* | [77] |
| 364 | 5,7-dimethyl-1H-indazole | C_9_H_10_N_2_ | *E*. *ciliata* | [66] |
| 365 | 17a(H)-21b(H)-hopane | C_30_H_52_ | *E*. *ciliata* | [66] |
| 366 | 17b(H)-21a(H)-30-norhopane | C_29_H_50_ | *E*. *ciliata* | [66] |
| 367 | (22z)-stigmasta-5,22-dien-3b-ol, acetate | C_31_H_50_O_2_ | *E*. *ciliata* | [66] |
| 368 | 2-pentanol propanoate | C_8_H_16_O_2_ | *E*. *ciliata* | [66] |
| 369 | 2-ethyl-1H-benzimidazole | C_9_H_10_N_2_ | *E*. *ciliata* | [66] |
| 370 | neophytadiene | C_20_H_38_ | *E*. *ciliata* | [66] |
| 371 | benzothiazole | C_7_H_5_NS | *E*. *ciliata* | [66] |
| 372 | β-sitosterol acetates | C_31_H_52_O_2_ | *E*. *ciliata* | [66] |
| 373 | farnesene-3-ol | C_15_H_26_O | *E*. *ciliata* | [66] |
| 374 | fluorene | C_13_H_10_ | *E*. *ciliata* | [66] |
| 375 | fucosteral | C_29_H_48_O | *E*. *ciliata* | [66] |
| 376 | decanoic acid, methyl ester | C_11_H_22_O_2_ | *E*. *ciliata* | [66] |
| 377 | indene | C_9_H_8_ | *E*. *ciliata* | [66] |
| 378 | L-linalool | C_10_H_18_O | *E*. *ciliata* | [66] |
| 379 | norprista-2-one | C_18_H_36_O | *E*. *ciliata* | [66] |
| 380 | octen-1-ol, acetate | C_10_H_18_O_2_ | *E*. *ciliata* | [66] |
| 381 | phytadiene | C_20_H_38_ | *E*. *ciliata* | [66] |
| 382 | phytane | C_20_H_42_ | *E*. *ciliata* | [66] |
| 383 | pristane | C_19_H_40_ | *E*. *ciliata* | [66] |
| 384 | propanoic acid butyl ester | C_7_H_14_O_2_ | *E*. *ciliata* | [66] |
| 385 | stigmast-4-en-3-one | C_29_H_48_O | *E*. *ciliata* | [66] |
| 386 | γ-sitosterol | C_29_H_50_O | *E*. *ciliata* | [66] |
| 387 | alloocimeme | C_10_H_16_ | *E*. *ciliata* | [67] |
| 388 | artemisia triene | C_10_H_16_ | *E*. *ciliata* | [67] |
| 389 | dihydrotagetone | C_10_H_18_O | *E*. *ciliata* | [67] |
| 390 | E,E-a-farnesene | C_15_H_24_ | *E*. *ciliata* | [67] |
| 391 | 1,4-pentadiene | C_5_H_8_ | *E*. *ciliata* | [67] |
| 392 | 1,6-germacradien-5-ol | C_15_H_26_O | *E*. *ciliata* | [67] |
| 393 | 1-undecanol | C_11_H_24_O | *E*. *ciliata* | [67] |
| 394 | β-farnesene | C_15_H_24_ | *E*. *ciliata* | [67] |
|  |  |  | *E*. *polystachya* | [70] |
|  |  |  | *E*. *stauntonii* | [71] |
| 395 | 1,4-terpineol | C_10_H_18_O | *E*. *rugulosa* | [68] |
| 396 | 1-cyclopenten-3-ol | C_5_H_8_O | *E*. *rugulosa* | [68] |
| 397 | 2-methyl-3-buten-2-ol | C_5_H_10_O | *E*. *rugulosa* | [68] |
| 398 | 3,5,5-trimethyl-2-cyclopenten-1-one | C_8_H_12_O | *E*. *rugulosa* | [68] |
| 399 | 4,5-dimethyl octane | C_10_H_22_ | *E*. *rugulosa* | [68] |
| 400 | 4,4-dimethyl-2-pentene | C_7_H_14_ | *E*. *rugulosa* | [68] |
| 401 | 5-methyl-2-furancarboxaldehyde | C_6_H_6_O_2_ | *E*. *rugulosa* | [68] |
| 402 | β-thujene | C_10_H_16_ | *E*. *rugulosa* | [68] |
| 403 | piperitol | C_10_H_18_O | *E*. *rugulosa* | [68] |
| 404 | p-menth-1(7)-en-9-ol | C_10_H_18_O | *E*. *rugulosa* | [68] |
| 405 | trimethyl phenylethylnyl silane | C_10_H_18_Si | *E*. *rugulosa* | [68] |
| 406 | nerolidol | C_15_H_26_O | *E*. *rugulosa* | [68] |
| 407 | 1,7,7-trimethyl-2-norbornene | C_10_H_16_ | *E*. *enistacha* | [69] |
| 408 | 2-methyl propenal | C_6_H_12_O | *E*. *enistacha* | [69] |
| 409 | 2-ethyl furan | C_6_H_10_O | *E*. *enistacha* | [69] |
| 410 | 2-hexenal | C_6_H_10_O | *E*. *enistacha* | [69] |
| 411 | 2-pentene | C_5_H_10_ | *E*. *enistacha* | [69] |
| 412 | 4-isopropyl benzaldehyde | C_10_H_12_O | *E*. *enistacha* | [69] |
| 413 | α-limonene | C_10_H_16_ | *E*. *enistacha* | [69] |
| 414 | β-citral | C_10_H_16_O | *E*. *enistacha* | [69] |
| 415 | bicycloelemene | C_15_H_24_ | *E*. *enistacha* | [69] |
| 416 | isolongifolene | C_15_H_24_ | *E*. *enistacha* | [69] |
| 417 | perillaldehyde | C_10_H_14_O | *E*. *enistacha* | [69] |
| 418 | spiro [4,5] decan-1-one | C_10_H_16_O | *E*. *enistacha* | [69] |
| 419 | γ-calarene | C_15_H_24_ | *E*. *enistacha* | [69] |
| 420 | 1,4-cineole | C_10_H_18_O | *E*. *polystachya* | [70] |
| 421 | α-longipinene | C_15_H_24_ | *E*. *polystachya* | [70] |
| 422 | α-p-dimethyl styrene | C_10_H_12_ | *E*. *polystachya* | [70] |
| 423 | 3,7-dimethyl-2,7-octadien-1 yl propionate | C_13_H_22_O_2_ | *E*. *polystachya* | [70] |
| 424 | 7-methyl-3-methylene-7-octen-1-yl propionate | C_13_H_22_O_2_ | *E*. *polystachya* | [70] |
| 425 | α-ylangene | C_15_H_24_ | *E*. *polystachya* | [70] |
| 426 | calamenene | C_15_H_22_ | *E*. *polystachya* | [70] |
| 427 | cis-ocimene | C_10_H_16_ | *E*. *polystachya* | [70] |
| 428 | irridomyrmecin | C_10_H_12_ | *E*. *polystachya* | [70] |
| 429 | δ-selinene | C_15_H_24_ | *E*. *polystachya* | [70] |
| 430 | γ-murolene | C_15_H_24_ | *E*. *stauntonii* | [71] |
| 431 | longiborn-8-ene | C_15_H_24_ | *E*. *stauntonii* | [71] |
| 432 | palmitic acid ethyl ester | C_18_H_36_O_2_ | *E*. *stauntonii* | [71] |
| 433 | patchouli alcohol | C_15_H_26_O | *E*. *stauntonii* | [71] |
| 434 | α-elemene | C_15_H_24_ | *E*. *stauntonii* | [71] |
| 435 | 1a, 9a-dimethyl-cis-bicyclo [4,3,0] non-7-en-2-one | C_11_H_16_O | *E*. *stauntonii* | [71] |
| 436 | bornylene | C_10_H_16_ | *E*. *stauntonii* | [71] |
| 437 | α-methylbenzenepropanol | C_10_H_14_O | *E*. *bodinieri* | [72] |
| 438 | β-elemenone | C_15_H_22_O | *E*. *bodinieri* | [72] |
| 439 | ethyl palmitate | C_18_H_36_O_2_ | *E*. *bodinieri* | [72] |
| 440 | eremophilene | C_15_H_24_ | *E*. *bodinieri* | [72] |
| 441 | dibuty phthalate | C_16_H_22_O_4_ | *E*. *bodinieri* | [72] |
| 442 | tran-β-farnesene | C_15_H_24_ | *E*. *bodinieri* | [72] |
| 443 | trans-3-hexen-1-ol | C_6_H_12_O | *E*. *splendens* | [73] |
| 444 | methyl eugenol | C_11_H_14_O_2_ | *E*. *splendens* | [73] |
| 445 | jasmone | C_11_H_16_O | *E*. *splendens* | [73] |
| 446 | naginata ketone | C_10_H_12_O_2_ | *E*. *splendens* | [73] |
| 447 | safrole | C_10_H_10_O_2_ | *E*. *splendens* | [73] |
| 448 | δ-cadinol | C_15_H_26_O | *E*. *splendens* | [73] |
| 449 | 1-octen-5-ol | C_8_H_16_O | *E*. *splendens* | [73] |
| 450 | 2,6,10,14-tertra-methyl-hexadecane | C_20_H_42_ | *E*. *splendens* | [73] |
| 451 | β-bourbonene | C_15_H_24_ | *E*. *splendens* | [73] |
|  |  |  | *E*. *bodinieri* | [75] |
| 452 | 1,3,6,10-dodecatetraene, 3,7,11-trimethyl-(Z,E)- | C_15_H_24_ | *E*. *bodinieri* | [75] |
| 453 | 1aR-(1aα., 4β, 4aβ, 7β, 7aβ,7b. α)]-decahydro-1,1,4,7- tetramethyl,  1H-cycloprop [e] azulene | C_15_H_26_ | *E*. *bodinieri* | [75] |
| 454 | 1-cyclohexylheptene | C_13_H_24_ | *E*. *bodinieri* | [75] |
| 455 | 1-iodohexadecane | C_16_H_33_I | *E*. *bodinieri* | [75] |
| 456 | 1-methyl-4-(1-methylethyl)- 1,4-cyclohexadiene | C_10_H_16_ | *E*. *bodinieri* | [75] |
| 457 | 1-methyl-5-methylene-8-(1-isopropyl)-1, 6-cyclodecadiene | C_15_H_24_ | *E*. *bodinieri* | [75] |
| 458 | 1-oxobutyl-1, 2-dihydropyridin | C_9_H_13_NO | *E*. *bodinieri* | [75] |
| 459 | (1R)-camphanone | C_10_H_16_O | *E*. *bodinieri* | [75] |
| 460 | 2,6-dimethyl-2,4-heptadiene | C_9_H_16_ | *E*. *bodinieri* | [75] |
| 461 | 4-(1-methylethyl)-benzene ethanol | C_11_H_16_O | *E*. *bodinieri* | [75] |
| 462 | 3-methyl-2-(5H)-furanone | C_5_H_6_O_2_ | *E*. *bodinieri* | [75] |
| 463 | 4-(2,7,7,-trimethyl bicyclo[3.2.0]-2-heptylene)-acetyl ketene | C_15_H_20_O | *E*. *bodinieri* | [75] |
| 464 | 4-hydroxy-3,5-dimethyl-6-(4-(2-methyl-3-(p-nitrophenyl)-2- propenylidene)  tetrahydro-2-furyl)-2-pyranone | C_21_H_21_NO_6_ | *E*. *bodinieri* | [75] |
| 465 | 5-methyl-2-isopropyl-9-methylene bicyclo[4.4.0] decene | C_15_H_24_ | *E*. *bodinieri* | [75] |
| 466 | cis-α-bisabolene epoxide | C_15_H_24_O | *E*. *bodinieri* | [75] |
| 467 | cis-epoxylinalol | C_10_H_18_O_2_ | *E*. *bodinieri* | [75] |
| 468 | cis-nerolidol | C_15_H_26_O | *E*. *bodinieri* | [75] |
| 469 | dehydroelsholtzia ketone | C_10_H_12_O_2_ | *E*. *bodinieri* | [75] |
| 470 | hexadecatrienoic acid methyl ester | C_17_H_28_O_2_ | *E*. *bodinieri* | [75] |
| 471 | hexahydrofarnesylacetone | C_18_H_36_O | *E*. *bodinieri* | [75] |
| 472 | myristic acid | C_17_H_34_O_2_ | *E*. *bodinieri* | [75] |
| 473 | nonacosane | C_29_H_60_ | *E*. *bodinieri* | [75] |
| 474 | selinene | C_15_H_24_ | *E*. *bodinieri* | [75] |
| 475 | tetracosane | C_24_H_50_ | *E*. *bodinieri* | [75] |
| 476 | p-cymene | C_10_H_14_ | *E*. *bodinieri* | [75] |
| 477 | (R)-4-methyl-1-(1-methylethyl)-3-cyclohexene-1-ol terpinen-4-ol | C_10_H_18_O | *E*. *bodinieri* | [76] |
| 478 | S(+)-5-(1-hydroxyl-1-methylethyl)-2-methyl-2-cyclohexene −1-one | C_10_H_18_O_3_ | *E*. *bodinieri* | [76] |
| 479 | trans-p-mentha-2,8-dienol | C_10_H_16_O | *E*. *bodinieri* | [76] |
| 480 | ledol | C_15_H_26_O | *E*. *bodinieri* | [76] |
| 481 | 1R-(1R*,4z,9S*)]-4,11,11-trimethyl-8-methylene-bicyclo [7.2.0]undec-4-ene | C_15_H_24_ | *E*. *bodinieri* | [76] |
| 482 | [1S-(1α,3α,5α)]-6,6-dimethyl-2-methylene-bicyclo[3.1.1] hexane-3-ol | C_11_H_18_O | *E*. *bodinieri* | [76] |
| 483 | 2-methyl-2-(3-methyl-2-oxobutyl)-1-cyclohexanone | C_12_H_20_O_2_ | *E*. *bodinieri* | [76] |
| 484 | 3-methylene-*p*-mentha-8-ene | C_11_H_18_ | *E*. *bodinieri* | [76] |
| 485 | 4-(2,2,6-Trimethylbicyclo[4.1.0]hept-1-yl)-2-butanone | C_14_H_24_O | *E*. *bodinieri* | [76] |
| 486 | 4,6,6-trimethyl-bicyclo[3.1.1]hept-3-ene-2-one | C_10_H_14_O | *E*. *bodinieri* | [76] |
| 487 | bicyclo (3.1.0)hexan-2-one, 5-(1-methylethyl)- | C_9_H_14_O | *E*. *bodinieri* | [76] |
| 488 | 10-undecenyl acetate | C_13_H_24_O_2_ | *E*. *bodinieri* | [76] |
| 489 | 11-hexadecyn-1-ol | C_16_H_30_O | *E*. *bodinieri* | [76] |
| 490 | α,α,4-trimethyl-(R)-3-cyclohexene-1-methanol acetate | C_12_H_20_O_2_ | *E*. *bodinieri* | [76] |
| 491 | decyl acetate | C_12_H_24_O_2_ | *E*. *bodinieri* | [76] |
| 492 | (E)-3(10)-caren-2-ol | C_10_H_16_O | *E*. *bodinieri* | [76] |
| 493 | 2-dodecanone | C_12_H_24_O | *E*. *stauntonii* | [77] |
| 494 | 2-methyl-2-butenolide | C_5_H_6_O_2_ | *E*. *stauntonii* | [77] |
| 495 | 1-hexanol-2-ethyl | C_8_H_18_O | *E*. *stauntonii* | [77] |
| 496 | 1[10],6,8-triene-cadina | C_15_H_22_ | *E*. *stauntonii* | [77] |
| 497 | 4,5.6,7,8,8a-hexahydro-8a-methyl-2[1H]-azuleneone | C_11_H_16_O | *E*. *stauntonii* | [77] |
| 498 | benzeneacetadehyde, a-ethylidene- | C_10_H_10_O | *E*. *stauntonii* | [77] |
| 499 | β-linalool | C_10_H_18_O | *E*. *stauntonii* | [77] |
| 500 | isoborneol | C_10_H_18_O | *E*. *stauntonii* | [77] |
| 501 | eudesmol | C_15_H_26_O | *E*. *stauntonii* | [77] |
| 502 | 7-octen-4-ol | C_8_H_16_O | *E*. *stauntonii* | [77] |
| 503 | 2,3-diethyl-6-methyl pyrazine | C_9_H_14_N_2_ | *E*. *ciliata* | [79] |
| 504 | 3-methoxy-6-ethynyl benzyl alcohol | C_10_H_10_O_2_ | *E*. *ciliata* | [79] |
| 505 | N-heptyildene methylamine | C_8_H_17_N | *E*. *ciliata* | [79] |
| 506 | 2,3-dimethyl-hexane | C_8_H_18_ | *E*. *splendens* | [106] |
| 507 | 2-pentenenitrile | C_5_H_7_N | *E*. *splendens* | [106] |
| 508 | 3-butanenitrile | C_4_H_7_N | *E*. *splendens* | [106] |
| 509 | 3-methyl-butanal | C_8_H_16_O | *E*. *splendens* | [106] |
| 510 | (1,3-diemethyl-2-methylene-cyclopentyl)-methanol | C_9_H_16_O | *E*. *argyi* | [114] |
| 511 | 1-methyl-5-(1-methylethenyl)-cyclohexene | C_10_H_16_ | *E*. *argyi* | [114] |
| 512 | [s-(E,E)]-1-methyl-5-methylene-8-(1-isopropyl)-1,6- cyclodecadiene | C_15_H_24_ | *E*. *argyi* | [114] |
| 513 | 2,6-dimethyl-6-(4-methyl-3-pentenyl)-bicyclo [3.1.1]hept-2-ene | C_15_H_24_ | *E*. *argyi* | [114] |
| 514 | 2,6,11,15-tertramethyl-hexadeca-2,6,8,10,14-pentane | C_20_H_32_ | *E*. *argyi* | [114] |
| 515 | 2,6-octadienoic acid, 3,7-dimethyl-methyl ester | C_11_H_18_O_2_ | *E*. *argyi* | [114] |
| 516 | (Z)-2,6-octadien-1-ol, 3,8-dimethyl-acetate | C_12_H_20_O_2_ | *E*. *argyi* | [114] |
| 517 | 2,3-dimethyl-5-(2,6,10-trimethylundecyl) furan | C_20_H_36_O | *E*. *argyi* | [114] |
| 518 | 4,4-dimethyl tetracyclo [6.3.2.0 (2,5).0(1,8)] tridecan-9-ol | C_15_H_24_O | *E*. *argyi* | [114] |
| 519 | 2,6,10-trimethyl dodecane | C_15_H_32_ | *E*. *argyi* | [114] |
| 520 | 3,7-dimethyl-(E)-2,6-octadienal | C_10_H_16_O | *E*. *argyi* | [114] |
| 521 | 3,7-dimethyl-(Z)-2,6-octadienal | C_10_H_16_O | *E*. *argyi* | [114] |
| 522 | (Z)-7,11-dimethyl-3-methylene-1,6,10-dodecatrinene | C_15_H_24_ | *E*. *argyi* | [114] |
| 523 | 9,12,15-octadecatrienoic acid, 2-phenyl-1,3-dioxan-5-yl ester | C_28_H_40_O_4_ | *E*. *argyi* | [114] |
| 524 | E-farnesene epoxide | C_15_H_24_O | *E*. *argyi* | [114] |
| 525 | geranic acid | C_10_H_16_O_2_ | *E*. *argyi* | [114] |
| 526 | trans-α-bergamotene | C_15_H_24_ | *E*. *argyi* | [114] |
| 527 | 6,10,14-trimethyl-2-pentadecanone | C_18_H_36_O | *E. stauntonii* | [47] |
|  |  |  | *E*. *bodinieri* | [48,76] |
|  |  |  | *E*. *argyi* | [114] |
| 528 | 3-octanol | C_8_H_18_O | *E*. *calycoarpa* | [53] |
|  |  |  | *E*. *feddei* | [54] |
|  |  |  | *E*. *frulicosa* | [57] |
|  |  |  | *E*. *densa* | [58] |
|  |  |  | *E*. *ciliata* | [65,79] |
|  |  |  | *E*. *splendens* | [73] |
|  |  |  | *E*. *stauntonii* | [77] |
|  |  |  | *E*. *argyi* | [114] |
| 529 | 1,5,5,8-tetramethyl-12-oxabicyclo [9.1.0] dodeca-3,7-diene | C_15_H_24_O | *E*. *stauntonii* | [77] |
|  |  |  | *E*. *argyi* | [114] |
| 530 | 1,1-diethyoxy-ethane | C_6_H_14_O_2_ | *E*. *cypriani* | [115] |
| 531 | 1-(2-furanyl)-ethanone | C_6_H_6_O_2_ | *E*. *cypriani* | [115] |
| 532 | 1,2-dimethyl benyene | C_8_H_10_ | *E*. *cypriani* | [115] |
| 533 | 1,(6);2,(3)-dianhydro-4-O-acetyl-β-D-mannopyranose | C_8_H_10_O_5_ | *E*. *cypriani* | [115] |
| 534 | 1-methyl-2-(1-methyl ethyl)-benzene | C_10_H_14_ | *E*. *cypriani* | [115] |
| 535 | 2,2-dimethyl-1,3-dioxolane | C_5_H_10_O_2_ | *E*. *cypriani* | [115] |
| 536 | 2,3-dihydro benzofuran | C_8_H_8_O | *E*. *cypriani* | [115] |
| 537 | 2-methylene-4,8,8-trimethyl-4-vinyl-bicyclo [5,2,0] nonane | C_15_H_24_ | *E*. *cypriani* | [115] |
| 538 | 3,7-dimethyl-1,3,6-octatriene | C_10_H_16_ | *E*. *cypriani* | [115] |
| 539 | 5-ethenyltetrhydro-α, α,5-trimethyl-2-furanmethanol | C_11_H_18_O_2_ | *E*. *cypriani* | [115] |
| 540 | 5-methyl furfural | C_6_H_6_O_2_ | *E*. *cypriani* | [115] |
| 541 | acetic acid | C_2_H_4_O_2_ | *E*. *cypriani* | [115] |
| 542 | benzene acetadehyde | C_8_H_8_O | *E*. *cypriani* | [115] |
| 543 | β-methyl-benzenepropanol | C_10_H_12_O | *E*. *cypriani* | [115] |
| 544 | decahydro naphthalene | C_10_H_18_ | *E*. *cypriani* | [115] |
| 545 | dihydro-2-methyl-3(2H) furanone | C_5_H_8_O_2_ | *E*. *cypriani* | [115] |
| 546 | ethyl propanonate | C_5_H_10_O_2_ | *E*. *cypriani* | [115] |
| 547 | furan | C_4_H_4_O | *E*. *cypriani* | [115] |
| 548 | n-propyl acetate | C_5_H_10_O_2_ | *E*. *cypriani* | [115] |
| 549 | phenol | C_6_H_6_O | *E*. *cypriani* | [115] |
| 550 | α-terpineol | C_10_H_18_O | *E. stauntonii* | [47,59,71,77] |
|  |  |  | *E*. *penduliflora* | [52] |
|  |  |  | *E*. *frulicosa* | [57,112] |
|  |  |  | *E*. *densa* | [58] |
|  |  |  | *E*. *strobilifera* | [61] |
|  |  |  | *E*. *blanda* | [63] |
|  |  |  | *E*. *ciliata* | [65,66] |
|  |  |  | *E*. *cypriani* | [115] |
| 551 | α-caryophyllene | C_15_H_24_ | *E*. *feddei* | [54] |
|  |  |  | *E*. *stauntonii* | [60] |
|  |  |  | *E*. *ciliata* | [65,66] |
|  |  |  | *E*. *cypriani* | [115] |
| 552 | acetophenone | C_8_H_8_O | *E*. *feddei* | [54] |
|  |  |  | *E*. *densa* | [58] |
|  |  |  | *E*. *stauntonii* | [59,60,71,77] |
|  |  |  | *E*. *strobilifera* | [61] |
|  |  |  | *E*. *blanda* | [50,63] |
|  |  |  | *E*. *splendens* | [73] |
|  |  |  | *E*. *bodinieri* | [75] |
|  |  |  | *E*. *ciliata* | [66,79] |
|  |  |  | *E*. *argyi* | 114] |
|  |  |  | *E*. *cypriani* | [115] |
| 553 | 3-octanone | C_8_H_16_O | *E*. *frulicosa* | [57] |
|  |  |  | *E*. *ciliata* | [66,79] |
|  |  |  | *E*. *cypriani* | [115] |
| 554 | carvacryl acetate | C_12_H_16_O_2_ | *E*. *ciliata* | [65] |
|  |  |  | *E*. *cypriani* | [115] |
| 555 | carvacrol | C_10_H_14_O | *E*. *densa* | [47,58] |
|  |  |  | *E*. *bodinieri* | [48,72] |
|  |  |  | *E*. *penduliflora* | [52] |
|  |  |  | *E*. *splendens* | [56] |
|  |  |  | *E*. *frulicosa* | [57] |
|  |  |  | *E*. *strobilifera* | [61] |
|  |  |  | *E*. *ciliata* | [65,66] |
|  |  |  | *E*. *cypriani* | [115] |
| 556 | 1-octen-3-ol | C_8_H_16_O | *E*. *bodinieri* | [49] |
|  |  |  | *E*. *penduliflora* | [52] |
|  |  |  | *E*. *stauntonii* | [60] |
|  |  |  | *E*. *ciliata* | [65] |
|  |  |  | *E*. *argyi* | [114] |
|  |  |  | *E*. *cypriani* | [115] |
| 557 | camphor | C_10_H_16_O | *E*. *blanda* | [50,62,63] |
|  |  |  | *E*. *ciliata* | [65,66] |
|  |  |  | *E*. *splendens* | [73] |
|  |  |  | *E*. *stauntonii* | [71,77] |
|  |  |  | *E*. *cypriani* | [115] |
| 558 | (−)-trans-pinocarvyl acetate | C_12_H_18_O_2_ | *E*. *frulicosa* | [116] |
| 559 | 1-octanone | C_8_H_16_O | *E*. *frulicosa* | [116] |
| 560 | 1-octanol | C_8_H_18_O | *E*. *frulicosa* | [116] |
| 561 | 1-methoxyl-naphthalene | C_11_H_10_O | *E*. *frulicosa* | [116] |
| 562 | 1-octen-3-ol | C_8_H_16_O | *E*. *frulicosa* | [116] |
| 563 | 1R-α-pinene | C_10_H_16_ | *E*. *frulicosa* | [116] |
| 564 | 2-pentyl-cyclopent-2-en-1-one | C_10_H_16_O | *E*. *frulicosa* | [116] |
| 565 | 9,12-octadecadienoic acid methyl ester | C_19_H_34_O_2_ | *E*. *frulicosa* | [116] |
| 566 | 9-epi-(E)-caryophyllene | C_15_H_24_ | *E*. *frulicosa* | [116] |
| 567 | 14-hydroxyl-9-epi-(E)-caryophyllene | C_15_H_24_O | *E*. *frulicosa* | [116] |
| 568 | α-amorphene | C_15_H_24_ | *E*. *frulicosa* | [116] |
| 569 | α-bisabolene | C_15_H_24_ | *E*. *frulicosa* | [116] |
| 570 | α-curcumene | C_15_H_22_ | *E*. *frulicosa* | [116] |
| 571 | β-damascenone | C_13_H_18_O | *E*. *frulicosa* | [116] |
| 572 | butanoic acid, 2-methyl-,2-methylbutylester | C_10_H_20_O_2_ | *E*. *frulicosa* | [116] |
| 573 | cadinol | C_15_H_26_O | *E*. *frulicosa* | [116] |
| 574 | cis-pinocarvyl acetate | C_12_H_18_O_2_ | *E*. *frulicosa* | [116] |
| 575 | cubenol | C_15_H_26_O | *E*. *frulicosa* | [116] |
| 576 | cyclopentadecanolide | C_15_H_28_O_2_ | *E*. *frulicosa* | [116] |
| 577 | geraniol formate | C_11_H_18_O_2_ | *E*. *frulicosa* | [116] |
| 578 | guaiol | C_15_H_26_O | *E*. *frulicosa* | [116] |
| 579 | isomenthol | C_10_H_20_O | *E*. *frulicosa* | [116] |
| 580 | isophytol | C_20_H_40_O | *E*. *frulicosa* | [116] |
| 581 | L-β-pinene | C_10_H_16_ | *E*. *frulicosa* | [116] |
| 582 | octen-3-yl, acetate | C_10_H_18_O_2_ | *E*. *frulicosa* | [116] |
| 583 | pentadecanoic acid | C_15_H_30_O_2_ | *E*. *frulicosa* | [116] |
| 584 | phytol | C_20_H_40_O | *E*. *frulicosa* | [116] |
| 585 | viridiflorene | C_15_H_24_ | *E*. *frulicosa* | [116] |
| 586 | γ-curcumene | C_15_H_22_ | *E*. *frulicosa* | [116] |
| 587 | γ-himachalene | C_15_H_24_ | *E*. *frulicosa* | [116] |
| 588 | γ-terpineol | C_10_H_18_O | *E*. *frulicosa* | [116] |
| 589 | tetradecanoic acid | C_14_H_28_O_2_ | *E*. *frulicosa* | [116] |
| 590 | β-trans-ocimene | C_10_H_16_ | *E*. *frulicosa* | [116] |
| 591 | terpene-4-ol | C_10_H_18_O | *E*. *frulicosa* | [116] |
| 592 | α-cedrene | C_15_H_24_ | *E*. *splendens* | [55] |
|  |  |  | *E*. *frulicosa* | [116] |
| 593 | 1,8-cineole | C_10_H_18_O | *E*. *stauntonii* | [47,59,71] |
|  |  |  | *E*. *penduliflora* | [52] |
|  |  |  | *E*. *frulicosa* | [57,116] |
|  |  |  | *E*. *strobilifera* | [61] |
|  |  |  | *E*. *blanda* | [50,62,63] |
|  |  |  | *E*. *polystachya* | [70] |
|  |  |  | *E*. *splendens* | [73] |
|  |  |  | *E*. *ciliata* | [79] |
| 594 | β-bisabolene | C_15_H_24_ | *E*. *bodinieri* | [48] |
|  |  |  | *E*. *splendens* | [56] |
|  |  |  | *E*. *strobilifera* | [61] |
|  |  |  | *E*. *patrini* | [64] |
|  |  |  | *E*. *bodinieri* | [72] |
|  |  |  | *E*. *frulicosa* | [116] |
| 595 | α-phellandrene | C_10_H_16_ | *E*. *bodinieri* | [48,72] |
|  |  |  | *E*. *blanda* | [50] |
|  |  |  | *E*. *calycoarpa* | [53] |
|  |  |  | *E*. *splendens* | [56] |
|  |  |  | *E*. *frulicosa* | [57,116] |
|  |  |  | *E*. *stauntonii* | [59] |
|  |  |  | *E*. *strobilifera* | [61] |
|  |  |  | *E*. *ciliata* | [65] |
| 596 | α-thujene | C_10_H_16_ | *E*. *blanda* | [50] |
|  |  |  | *E*. *splendens* | [55] |
|  |  |  | *E*. *enistacha* | [69] |
|  |  |  | *E*. *polystachya* | [70] |
|  |  |  | *E*. *frulicosa* | [116] |
| 597 | α-humulene | C_15_H_24_ | *E*. *calycoarpa* | [53] |
|  |  |  | *E*. *strobilifera* | [61] |
|  |  |  | *E*. *ciliata* | [67,79] |
|  |  |  | *E*. *stauntonii* | [71] |
|  |  |  | *E*. *frulicosa* | [116] |
| 598 | α-farnesene | C_15_H_24_ | *E*. *feddei* | [54] |
|  |  |  | *E*. *ciliata* | [66,67] |
|  |  |  | *E*. *enistacha* | [69] |
|  |  |  | *E*. *splendens* | [73] |
|  |  |  | *E*. *argyi* | [114] |
|  |  |  | *E*. *frulicosa* | [116] |
| 599 | β-humulene | C_15_H_24_ | *E*. *polystachya* | [70] |
|  |  |  | *E*. *frulicosa* | [116] |
| 600 | 2-methyl-3-furan carboxylic acid methyl ester | C_7_H_8_O_3_ | *E*. *rugulosa* | [117] |
| 601 | 2-methyoxy-4-methyl phenol | C_8_H_10_O_2_ | *E*. *rugulosa* | [117] |
| 602 | 2-methoxy phenol | C_7_H_8_O_2_ | *E*. *rugulosa* | [117] |
| 603 | 2-methyl-2-none-4-one | C_10_H_18_O | *E*. *rugulosa* | [117] |
| 604 | 2-methyl-2-pentanal | C_6_H_12_O | *E*. *rugulosa* | [117] |
| 605 | 2-methyl-4-(1,1-dimethylethyl)-phenol | C_11_H_16_O | *E*. *rugulosa* | [117] |
| 606 | 2-cyclohexen-1-one, 4-(3-hydroxy-1-butenyl)-3,5,5-trimethyl- | C_13_H_22_O_2_ | *E*. *rugulosa* | [117] |
| 607 | 3a,7a-dihydro-5-methyl-indene-1,7-(4H)-dione | C_10_H_10_O_2_ | *E*. *rugulosa* | [117] |
| 608 | 3-methyl-butenoic acid cyclobutyl ester | C_9_H_16_O_2_ | *E*. *rugulosa* | [117] |
| 609 | 4,4,8-trimethyltricyclo [6.3.1.0(1,5)]dodecane-2,9-diol | C_15_H_26_O | *E*. *rugulosa* | [117] |
| 610 | 6-isopropenyl-4,8a-dimethyl-1,2,3,5,6,7,8,8a-octahydro- naphthalen-2-ol | C_15_H_24_O | *E*. *rugulosa* | [117] |
| 611 | 6-isopropenyl-4,8a-dimethyl-4a,5,6,7,8,8a-hexahydro-1H-naphthalen-2-one | C_15_H_22_O | *E*. *rugulosa* | [117] |
| 612 | 9,12,15-octadecadtien-1-ol, (Z,Z,Z)- | C_18_H_32_O | *E*. *rugulosa* | [117] |
| 613 | 7-acetyl-2-hydroxy-2-hydroxy-2-methyl-5-isopropylbicyclo [4.3.0] nonane | C_15_H_26_O_2_ | *E*. *rugulosa* | [117] |
| 614 | benzyl alcohol | C_7_H_8_O | *E*. *rugulosa* | [117] |
| 615 | cyclobuta [1,2:3,4] dicyclopentene, decahydro-3a-methyl-6- methylene-1-(1-methylethyl)-[1S(1α,3aα,3bβ,6aβ,6bα)]- | C_15_H_24_ | *E*. *rugulosa* | [117] |
| 616 | n-hexadecanoic acid | C_16_H_32_O_2_ | *E*. *rugulosa* | [117] |
| 617 | β-elemene | C_15_H_24_ | *E*. *bodinieri* | [48,72] |
|  |  |  | *E*. *feddei* | [54] |
|  |  |  | *E*. *blanda* | [63] |
|  |  |  | *E*. *patrini* | [64] |
|  |  |  | *E*. *rugulosa* | [68,117] |
|  |  |  | *E*. *polystachya* | [70] |
| 618 | α-cadinol | C_15_H_26_O | *E*. *bodinieri* | [48] |
|  |  |  | *E*. *splendens* | [55] |
|  |  |  | *E*. *stauntonii* | [60] |
|  |  |  | *E*. *bodinieri* | [72] |
|  |  |  | *E*. *frulicosa* | [116] |
|  |  |  | *E*. *rugulosa* | [117] |
| 619 | caryophylene oxide | C_15_H_24_O | *E*. *stauntonii* | [47,71] |
|  |  |  | *E*. *bodinieri* | [48,72,75] |
|  |  |  | *E*. *blanda* | [51] |
|  |  |  | *E*. *feddei* | [54] |
|  |  |  | *E*. *frulicosa* | [57,116] |
|  |  |  | *E*. *patrini* | [64] |
|  |  |  | *E*. *splendens* | [73] |
|  |  |  | *E*. *argyi* | [114] |
|  |  |  | *E*. *rugulosa* | [117] |
| 620 | 3,7-dimethyl-1,6-octadien-3-ol | C_10_H_18_O | *E*. *frulicosa* | [57] |
|  |  |  | *E*. *argyi* | [114] |
|  |  |  | *E*. *rugulosa* | [117] |
| 621 | caryophyllene | C_15_H_24_ | *E*. *feddei* | [54] |
|  |  |  | *E*. *strobilifera* | [61] |
|  |  |  | *E*. *patrini* | [64] |
|  |  |  | *E*. *argyi* | [114] |
|  |  |  | *E*. *frulicosa* | [116] |
|  |  |  | *E*. *rugulosa* | [117] |
| 622 | α-gurjunene | C_15_H_24_ | *E*. *strobilifera* | [61] |
|  |  |  | *E*. *polystachya* | [70] |
|  |  |  | *E*. *rugulosa* | [117] |
| 623 | zingiberene | C_15_H_24_ | *E*. *strobilifera* | [61] |
|  |  |  | *E*. *rugulosa* | [117] |
| 624 | 2-acetyl-5-methylfuran | C_7_H_8_O_2_ | *E*. *stauntonii* | [77] |
|  |  |  | *E*. *ciliata* | [79] |
|  |  |  | *E*. *rugulosa* | [117] |
| 625 | 2-furan methanol | C_5_H_6_O_2_ | *E*. *cypriani* | [114] |
|  |  |  | *E*. *rugulosa* | [117] |
| 626 | 1,2-dimethoxy-4-(2-propenyl)-benzene | C_11_H_14_O_2_ | *E*. *splendens* | [118] |
| 627 | 1,2,3-trimethoxyl-5-[2-propenyl ]benzene | C_12_H_16_O_3_ | *E*. *splendens* | [118] |
| 628 | 1,2,4-trimethoxyl-5-[propenyl]benzene | C_12_H_16_O_3_ | *E*. *splendens* | [118] |
| 629 | 1,3,7,7-tetramethyl-9-oxo-2-oxabicyclo[4.4.0]-5-ene | C_13_H_20_O_2_ | *E*. *splendens* | [118] |
| 630 | 1-ethoxyl pentane | C_7_H_16_O | *E*. *splendens* | [118] |
| 631 | 2,3,4-trimethyl-1,3-dioxolane | C_6_H_12_O_2_ | *E*. *splendens* | [118] |
| 632 | 4,4,7a-trimethyl-5,6,7,7a-tetrahydro-2[4H]benzofuranone | C_11_H_16_O_2_ | *E*. *splendens* | [118] |
| 633 | 4,5-dimethyl-1,3-dioxolan-2-one | C_5_H_8_O_3_ | *E*. *splendens* | [118] |
| 634 | 4-methoxyl-6-[2-propenyl]-1,3-benzodioxole | C_11_H_12_O_3_ | *E*. *splendens* | [118] |
| 635 | 5-methyl-2-isopropyl phenol acetate | C_12_H_16_O_2_ | *E*. *splendens* | [118] |
| 636 | α,α-dimethyl phenylethyl acetate | C_12_H_16_O_2_ | *E*. *splendens* | [118] |
| 637 | S-ethyl lactate | C_5_H_10_O_3_ | *E*. *splendens* | [118] |
| 638 | E-ethyl lactate | C_5_H_10_O_3_ | *E*. *splendens* | [118] |
| 639 | 1-methyl-4-isopropyl benzene | C_10_H_14_ | *E*. *splendens* | [114] |
| 640 | 6-methyl-5-hepten-2-one | C_8_H_14_O | *E*. *blanda* | [51] |
|  |  |  | *E*. *strobilifera* | [61] |
|  |  |  | *E*. *stauntonii* | [71] |
|  |  |  | *E*. *argyi* | [114] |
|  |  |  | *E*. *splendens* | [118] |
| 641 | 6-methyl-5-hepten-2-one | C_8_H_14_O | *E*. *blanda* | [51] |
|  |  |  | *E*. *strobilifera* | [61] |
|  |  |  | *E*. *stauntonii* | [71] |
|  |  |  | *E*. *argyi* | [114] |
|  |  |  | *E*. *splendens* | [118] |
| 642 | nerolidol | C_15_H_26_O | *E*. *stauntonii* | [47,71,77] |
|  |  |  | *E*. *blanda* | [51] |
|  |  |  | *E*. *feddei* | [54] |
|  |  |  | *E*. *splendens* | [73,118] |
| 643 | benzaldehyde | C_7_H_6_O | *E*. *blanda* | [50] |
|  |  |  | *E*. *penduliflora* | [52] |
|  |  |  | *E*. *ciliata* | [65,79] |
|  |  |  | *E*. *stauntonii* | [71] |
|  |  |  | *E*. *bodinieri* | [75] |
|  |  |  | *E*. *frulicosa* | [116] |
|  |  |  | *E*. *rugulosa* | [117] |
|  |  |  | *E*. *splendens* | [118] |
| 644 | β-selinene | C_15_H_24_ | *E*. *blanda* | [50] |
|  |  |  | *E*. *ciliata* | [65] |
|  |  |  | *E*. *splendens* | [118] |
| 645 | 3-carene | C_10_H_16_ | *E*. *feddei* | [54] |
|  |  |  | *E*. *ciliata* | [65,66] |
|  |  |  | *E*. *enistacha* | [69] |
|  |  |  | *E*. *cypriani* | [115] |
|  |  |  | *E*. *splendens* | [118] |
| 646 | 3-methyl-2-pentanone | C_6_H_12_O | *E*. *splendens* | [106,118] |
